# Structural and functional analyses of nematode-derived antimicrobial peptides support the occurrence of direct mechanisms of worm-microbiota interactions

**DOI:** 10.1016/j.csbj.2024.04.019

**Published:** 2024-04-10

**Authors:** James Rooney, Esperanza Rivera-de-Torre, Ruizhe Li, Kevin Mclean, Daniel R.G. Price, Alasdair J. Nisbet, Andreas H. Laustsen, Timothy P. Jenkins, Andreas Hofmann, Somenath Bakshi, Ashraf Zarkan, Cinzia Cantacessi

**Affiliations:** a Department of Veterinary Medicine, University of Cambridge, Cambridge, United Kingdom; b Department of Biotechnology and Biomedicine, Technical University of Denmark, Lyngby, Denmark; c Department of Engineering, University of Cambridge, Cambridge, United Kingdom; d Moredun Research Institute, Penicuik Midlothian, United Kingdom; e Max Rubner-Institut, Federal Research Institute of Nutrition and Food, Kulmbach, Germany; f Melbourne Veterinary School, Faculty of Science, The University of Melbourne, Parkville, Victoria 3010, Australia; g Department of Genetics, University of Cambridge, Cambridge, United Kingdom

**Keywords:** Helminth-microbiome interactions, Antimicrobial peptides, Structural analyses, Time-lapse microfluidics, Recombinant expression, Saposin, Metridin-like ShK toxin, *Bacillus subtilis*

## Abstract

The complex relationships between gastrointestinal (GI) nematodes and the host gut microbiota have been implicated in key aspects of helminth disease and infection outcomes. Nevertheless, the direct and indirect mechanisms governing these interactions are, thus far, largely unknown. In this proof-of-concept study, we demonstrate that the excretory-secretory products (ESPs) and extracellular vesicles (EVs) of key GI nematodes contain peptides that, when recombinantly expressed, exert antimicrobial activity in vitro against *Bacillus subtilis*. In particular, using time-lapse microfluidics microscopy, we demonstrate that exposure of *B. subtilis* to a recombinant saposin-domain containing peptide from the ‘brown stomach worm’, *Teladorsagia circumcincta*, and a metridin-like ShK toxin from the ‘barber’s pole worm’, *Haemonchus contortus*, results in cell lysis and significantly reduced growth rates. Data from this study support the hypothesis that GI nematodes may modulate the composition of the vertebrate gut microbiota directly via the secretion of antimicrobial peptides, and pave the way for future investigations aimed at deciphering the impact of such changes on the pathophysiology of GI helminth infection and disease.

## Graphical Abstract

**Figure:**
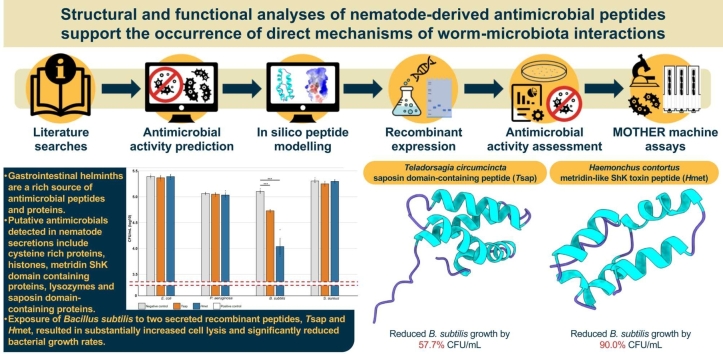

## 1 Introduction

Antimicrobial peptides (AMPs) are a ubiquitous and diverse group of peptides, typically less than 100 amino acid residues in length, whose cationic and amphipathic properties confer broad-spectrum antimicrobial activity against both prokaryotes and eukaryotes [1]. In particular, the antibacterial activity of AMPs is underpinned by several mechanisms, ranging from interference with the biosynthesis and processing of microbial DNA, RNA and proteins, to the disruption of membrane integrity through the formation of pores and/or channels that causes leakage of cellular components, or cell death by osmotic shock [2]. To date, over 3500 AMPs have been described from a wide range of vertebrate and invertebrate organisms, the latter including insects, crustaceans, spiders, molluscs, and free-living and parasitic worms (i.e., helminths) [3], [4], [5].

Helminths are amongst the most prevalent infectious agents of humans and animals worldwide [6]. Currently, around 1.5 billion people are estimated to be infected by helminths, causing over 3 million disability-adjusted life years (DALYs) annually, particularly in developing regions of the globe [6], [7]. Moreover, in Europe alone, helminths of livestock are estimated to cost the food industry around €1.8 billion/year due to animal death, productivity losses (e.g., reduced weight gain), and costs associated with veterinary intervention [8]. Current strategies of helminth control in both humans and livestock rely heavily on the administration of anthelmintics in mass drug administration (MDA) or in targeted strategic treatment (TST) programmes, respectively [9], [10]. However, resistance to all classes of anthelmintic compounds is now widespread in helminths of livestock, and the reliance on a relatively small number of anthelmintics in MDA programmes makes the emergence of drug-resistant human parasites both likely and concerning [11]. Thus, over the last decades, efforts have been directed towards achieving a better understanding of the fundamental biology of helminths and their interactions with their vertebrate hosts, with the ultimate goal of identifying novel targets for the development of sustainable strategies of parasite control [12].

One emerging area of interest in host-parasite interaction research is the elucidation of the crosstalk between helminths, particularly nematodes of the vertebrate gastrointestinal (GI) tract, and the resident gut microbiota/-me [13], [14], [15]. The latter is defined as *“a diverse consortium of bacteria, archaea, fungi, protozoa, viruses* [the microbiota]*, and their collective genome* [the microbiome]” [16], that play several key roles in host physiology, including nutrient absorption and metabolism, energy homeostasis, immune system development and defence against pathogens. Over the last decade, several studies, conducted in both humans and animals, under experimental and natural conditions of GI helminth infections, have consistently reported significant associations between worm colonisation and changes in the composition and/or function of the host gut microbiota [17], [18], [19]. However, the mechanisms governing worm-microbiota interactions remain, thus far, unclear [20]. Until recently, the vast majority of host-parasite interaction studies have focussed on the ability of helminths to communicate with and modulate the immune system of the host, particularly through the activity of their excretory/secretory products (ESPs) [21], [22]. Intriguingly, recent evidence points towards a role of helminth ESPs in worm-microbiota interactions, namely by exerting antimicrobial activity [5], [23], [24]. For instance, ESPs of the swine roundworm, *Ascaris suum,* have been shown to exert significant bactericidal activity against *Escherichia coli*, *Staphylococcus aureus,* and *Salmonella typhimurium*, and to disrupt biofilm formations of *E. coli* in vitro [5]. Indeed, *A. suum* ESPs contain several antimicrobial molecules, including cecropin-P2 [25], *A. suum* antibacterial factor (ASABF), lysozymes, as well as C-type lectin domain-containing proteins [5], that can disrupt bacterial membranes to cause cell lysis, or promote lethal agglutination of bacterial cells. More recently, we also demonstrated that in vitro exposure of *E. coli* to ESPs from the ‘brown stomach worm’, *Teladorsagia circumcincta*, an abomasal parasite of small ruminants, results in significant reductions of colony forming units over a 3-h period [24]. Proteomics analyses of *T. circumcincta* ESPs, coupled with bioinformatics screening of amino acid sequence data, identified several putative AMPs, including a histone H4-like protein, an *Ancylostoma*-secreted protein (ASP)-like protein, and an invertebrate-specific lysozyme-like enzyme known as destabilase [24]*.* Previously, we hypothesised that the antimicrobial properties of ESPs from *T. circumcincta*, as well as of ESPs from other GI helminths of humans and animals, may be attributed to the activity of secreted AMPs, free and/or encapsulated in secreted extracellular vesicles (EVs) [24]. Nevertheless, experimental evidence is essential to design follow-up mechanistic investigations aimed to translate fundamental knowledge of helminth-host-microbiota relationships into novel therapeutic approaches. Thus, in this study, we (i) mined publicly available ESP and EV proteomics sequence data for a range of GI nematodes of public health and veterinary importance and identified putative AMPs via targeted bioinformatics analyses; (ii) characterised structural and biochemical properties of selected AMP candidates; (iii) produced these candidates using heterologous expression in yeast; (iv) tested the activity of the resulting recombinant AMPs in vitro against representative Gram-negative and Gram-positive bacteria; and (v) performed microfluidics-based time-lapse microscopy to gather insights into their possible mode(s) of action. Of the putative AMPs identified across publicly available ESP and/or EV proteomics data from eight GI nematodes, two (i.e., a saposin domain-containing peptide from *T. circumcincta* EVs and a metridin ShK toxin domain-containing peptide from ESPs of the ‘barber’s pole worm’, *Haemonchus contortus*) exerted significant antibacterial activity against the Gram-positive *Bacillus subtilis*.

## 2 Methods

### 2.1 Literature search

Iterative literature searches were carried out to compile a list of publications linked to proteomics datasets from GI nematode ESP and/or EV material. In particular, Google Scholar (https://scholar.google.com/) and PubMed (https://pubmed.ncbi.nlm.nih.gov/) were interrogated using the search term “Gastrointestinal nematode excretory secretory products”. Several secondary searches were performed for each database targeting helminth species associated with qualitative and/or quantitative alterations in vertebrate gut microbiota composition according to published literature, e.g., “*Ascaris suum* excretory secretory products”, and “*Haemonchus contortus* excretory secretory products”. All searches were completed on or before 4th July 2023. From each search, articles that included fully accessible links to corresponding proteomics datasets, and/or up-to-date accession numbers for sequences identified during proteomics analysis, were compiled. For articles referring to the analysis of nematode ESP and/or EV proteomics data that were however inaccessible (unpublished or unobtainable), the corresponding author was contacted by email in an attempt to retrieve this information. A list of articles containing accession numbers linked to proteomics datasets was compiled. Accession numbers were subsequently extracted, uploaded onto the UniProt ID Mapping tool (https://www.uniprot.org/id-mapping; [26]), and corresponding amino acid sequences were retrieved. In instances where accession numbers could not be assigned to corresponding sequences using the UniProt ID Mapping tool, individual accession numbers were manually entered in the search string of the specific database cited in relevant publications. Accession numbers that could not be assigned protein sequences using either of the aforementioned methods were excluded from downstream analyses. Finally, all protein sequences and corresponding accession numbers were saved in FASTA format and subjected to AMP prediction analyses as described below. For *T. circumcincta*, the original proteomics datafiles obtained by Tzelos *et al.* [27] were used to interrogate a comprehensive in-house dataset of Iso-Seq transcripts from third- and fourth-stage larvae (L3 and L4, respectively) and adult male and female worms (data available from Mendeley data at DOI: 10.17632/hzrcpjh6y7.1). ESP and EV amino acid sequences identified using this approach were assigned InterPro identifiers (InterPro IDs) and Gene Ontology (GO) terms using OmicsBox (https://www.biobam.com/omicsbox) [28].

### 2.2 Antimicrobial peptide and protein prediction

All amino acid sequences collated as described above were screened for the occurrence of features indicative of likely antimicrobial activity [24]. Firstly, sequences were individually used to query the Collection of Anti-Microbial Peptides (CAMP_R3_) database (http://www.camp.bicnirrh.res.in/) [29]. Significant similarities between helminth amino acid sequences and AMPs in the CAMP_R3_ database were identified using the in-built BLASTp tool with the following criteria: matrix = BLOSUM62; alignment = ungapped; E-value threshold = 1E^−5^. In instances where a given query sequence shared significant similarity to multiple CAMP_R3_ sequences, only the top scoring hit was retained. Secondly, all GI helminth ESP- and EV-associated amino acid sequences were subjected to ampir analysis (https://ampir.marine-omics.net/). Ampir utilizes a supervised statistical machine learning approach to assign a probability score (ranging from 0 to 1, where 1 indicates the highest probability of antimicrobial activity), based on the physico-chemical properties of the amino acid patterns associated with each sequence. For each ESP- and EV-associated amino acid sequence, AMP prediction was performed using two vector machine classification models, i.e., “precursor” (best suited for analysis of full-length proteins) and “mature” (for sequences representing the final AMP after post-translational processing) [30]. The probability scores generated by each classification model were recorded; sequences returning ampir scores of > 0.7 were retained as ‘sequences of interest’. Finally, MultiPep (https://github.com/scheelelab/MultiPep) was used to predict the antimicrobial activity of each GI helminth ESP- and EV-associated amino acid sequence; MultiPep applies convolutional neural networks to assign each peptide to one of twenty bioactivity classes according to their intrinsic amino acid patterns [31]. Within each bioactivity class, each sequence is assigned a probability score ranging from 0 to 1, where 1 corresponds to the highest likelihood of accurately identifying a given query sequence as belonging to the respective bioactivity class. Since MultiPep screening is limited to sequences < 200 amino acids in length, sequences that did not meet this criterion were excluded from this analysis, while suitable amino acid sequences were formatted according to MultiPep requirements; the ‘MultiPep_predict.py’ Python script was executed as instructed by the software developer. For each of these sequences, bioactivity class scores were recorded; sequences with scores > 0.7 for either ‘antimicrobial’ or ‘antibacterial’ classes were retained as ‘sequences of interest’ [24].

GI nematode ESP- and EV-associated amino acid sequences that were marked as 'sequences of interest' using one or more antimicrobial activity prediction tools, and/or had been previously reported as exerting antimicrobial activity according to published literature, were compiled into a list of putative helminth-derived AMPs to be subjected to downstream analyses.

### 2.3 Peptide modelling

Computational structure prediction was performed for amino acid sequences with the highest probability of antimicrobial activity using Robetta (https://robetta.bakerlab.org/). Briefly, following removal of signal peptides, tertiary peptide models were generated using RoseTTAFold [32]. For each predicted model, the RoseTTAFold assigns a structure confidence score between 0 (low confidence) and 1 (high confidence). The highest confidence-scoring model was selected for downstream computational analyses and, for these models, the Protein Data Bank (PDB) coordinate file was obtained. The spatial arrangement of the predicted antimicrobial model at the bacterial membrane was simulated using the Positioning of Proteins in Membranes (PPM) 3.0 server (https://opm.phar.umich.edu/ppm_server3) in the Orientations of Proteins in Membranes (OPM) database (https://opm.phar.umich.edu/) [33] with the following parameters: (i) type of membrane = Gram-negative/-positive lipid bilayer membrane; (ii) allow curvature = no; (iii) include heteroatoms = no. PPM 3.0 outputs were saved, including the predicted position and orientation of the peptide of interest in the bacterial membrane, as well as the specific amino acid residues putatively involved in protein-membrane interactions (when applicable). The three-dimensional coordinates of the peptide-membrane assembly were downloaded as a PBD file and uploaded onto CHARMM-GUI (https://www.charmm-gui.org/) [34], where the bacterial membrane was rebuilt and subsequently visualised using ChimeraX (https://www.rbvi.ucsf.edu/chimerax/) [35].

The surface electrostatics of predicted peptide structures were analysed using the APBS-PDB2PQR software suite (https://server.poissonboltzmann.org/) [36]. First, predicted peptide model PDB coordinates were converted into PQR files using PDB2PQR with a PARSE forcefield [37]. The PQR files were subsequently uploaded to the Adaptive Poisson-Boltzmann Solver (APBS), and electrostatic surface values were generated. The output files were visualised on ChimeraX [35]. ChimeraX was also applied to the visualisation of any intra-peptide disulphide bonds and calculation of disulphide bond length. Thiol groups of pairs of cysteines within 2.0 Å and 3.0 Å were considered likely to form disulphide bonds [38]. For each peptide, the isoelectric point (pI), % amino acid composition, and overall charge at pH 7.4 were calculated using the Prot pi tool (https://www.protpi.ch/Calculator/ProteinTool). The probability of each peptide of interest forming homodimers was assessed using the Alphafold structure prediction tool [39] in combination with ColabFold [40], within ChimeraX [35]. Briefly, within ChimeraX, each putative helminth AMP sequence was used to query the Alphafold database, using default settings. For each query sequence, Alphafold generates five potential models, automatically selecting the ‘best’ model (i.e. that with highest confidence) based on predicted local distance difference test (pLDDT) scores and domain position confidence (PAE) values. For each sequence of interest, the ‘best’ predicted homodimer model was rendered in ChimeraX, with predicted pseudobonds (defined as “*a connection other than a covalent bond, such as a hydrogen bond, metal coordination bond*” by ChimeraX) between pairs of amino acid residues, > 5 Å apart, on separate peptide subunits. Pseudobonds were coloured according to PAE values.

### 2.4 Peptide expression

Recombinant expression of selected peptides of interest was carried out using the methilotrophic yeast *Komagataella phaffii* (previously known as *Pichia pastoris*) as the heterologous host system. In particular, following signal peptide removal, the codon usage of each sequence was optimised for expression in *K. phaffii* with the Genescript codon optimization tool [41] and the synthetised DNA sequence was ligated into the vector pPICZα A (Invitrogene), encoding zeocin resistance and an alpha secretion peptide from the α-mating factor from *Saccharomyces cerevisiae*, with an N-terminal His-tag and Tobacco Etch Virus (TEV) protease cleavage site. The vectors were electro-transformed into freshly prepared electrocompetent *K. phaffii* KM71H cells that were subsequently grown on YPDS plates (20 g/L peptone, 10 g/L yeast extract, 100 mL/L dextrose 20 % (w/v), 182.2 g/L sorbitol, 20 g/L agar) containing 1000 μg/mL zeocin over 72 h at 30 °C. Single colonies were selected and replated onto YPDS plates containing 1000 μg/mL zeocin. Individual colonies were inoculated into 10 mL of YPD (20 g/L peptone, 10 g/L yeast extract, 100 mL/L dextrose 20 % (w/v) in a 250 mL baffled flask and grown overnight at 30 °C with 250 rpm shaking. A 10 mL culture was used to inoculate 1 L of buffered glycerol complex medium (BMGY, 10 g/L yeast extract, 20 g/L peptone, 0.1 M potassium phosphate pH 6.0, 1.34 % (w/v) YNB, 0.04 µg/mL biotin, 1 % (v/v) glycerol) in a 5 L baffled flask and grown overnight at 30 °C with 250 rpm shaking, until the culture reached an OD_600_ between 2 and 6 as recommended by the manufacturer [42]. The cells were harvested by centrifugation at 3000 x g for 5 min at room temperature and subsequently resuspended into 200 mL of buffered methanol complex medium (BMMY, 10 g/L yeast extract, 20 g/L peptone, 0.1 M potassium phosphate pH 6.0, 1.34 % (w/v) YNB, 0.04 µg/mL biotin, 0.5 % (v/v) methanol) in a 1 L baffled flask, covered with 2 layers of sterile cheesecloth and incubated at 30 °C with constant shaking at 250 rpm. The culture was incubated for a total of 72 h, with 100 % methanol added to a final dilution of 0.5 % (v/v) every 24 h. The cells were then pelleted by centrifugation at 3000 x g for 5 min at room temperature, the supernatant was collected, sterilised by filtration with 0.2 µm filter (Millipore), and stored at 4 °C until further use.

Successfully expressed peptides were purified from the supernatant using Ni-NTA affinity chromatography in a gravity column setup, in which the Ni-NTA resin specifically binds to the N-terminal His-tag on the recombinant peptide. Briefly, the filtered supernatant was dialyzed against wash buffer (50 mM TRIS-HCl, pH 8.0, 300 mM NaCl, 20 mM imidazole) and mixed with 3 mL of HIS-Select® Nickel Affinity Gel resin (ThermoFisher) equilibrated in the same buffer. After 1 h of incubation at room temperature, the resin was loaded onto a column, the flow-through was collected, and the resin was washed with wash buffer (50 mM TRIS-HCl, pH 8.0, 300 mM NaCl, 20 mM imidazole), until the A_280_ of the column eluent reached < 0.05. The recombinant peptides were eluted with 5 column volumes of elution buffer (50 mM TRIS-HCl, pH 8.0, 300 mM NaCl, 400 mM imidazole) in 1 mL fractions. Subsequently, eluates containing peptides according to their A_280_ were pooled and dialysed at 4 °C against 1 L of PBS (pH 7.4) for at least 24 h. The dialysis buffer was refreshed 3 times during the dialysis process to ensure complete removal of the elution buffer. The concentration of each peptide was quantified using the Beer-Lambert law, with the absorbance reading taken at 280 nm and adjusted using the theoretical molar extinction coefficient of the peptide (https://web.expasy.org/protparam/) [43]. At each step of the purification process, peptide purity was verified using precast 4–12 % Novex Bis- TRIS sodium dodecyl sulphate-polyacrylamide gel electrophoresis (SDS-PAGE) gel (ThermoFisher) and stained with colloidal Coomassie Brilliant Blue (Sigma).

For Western blotting, protein lysates were first separated on an SDS-PAGE gel. Following separation, peptides were electrophoretically transferred to an Immobilon P membrane (Millipore). Once the transfer was completed, the membrane was blocked using 3 % milk in TBS containing 0.1 % Tween-20 (TBS-T) for 1 h at room temperature with gentle agitation. After blocking, the membrane was incubated with a primary anti-6His antibody (MA1–135, Invitrogen), diluted to a concentration of 1:250 in the blocking solution. This incubation was carried out overnight at 4 °C with gentle shaking. The following day, the primary antibody solution was discarded, and the membrane was washed three times for 10 min each using TBS-T. After the washes, the membrane was exposed to the secondary horse anti-mouse IgG (H+L) antibody conjugated to horseradish peroxidase (HRP) (PI-2000, Vector Laboratories), diluted to a 1:400 ratio in the blocking solution, for 1 h at room temperature with gentle agitation. Then, the membrane was again washed three times with TBS-T for 10 min. Finally, peptide bands were visualised using an Enhanced Chemiluminescence (ECL) system (Pierce, Thermofisher) and captured using a Biorad BioImager.

### 2.5 Antimicrobial activity assays

The bactericidal and/or bacteriostatic activity of each of the recombinantly expressed peptides was assessed against representative Gram-positive and -negative bacterial strains, i.e., *E. coli* BW25113 wild-type, *Pseudomonas aeruginosa* PA14*, B. subtilis* JH642*,* and *S. aureus* NCTC 12493 using a colony counting method [24]. Briefly, each bacterial strain was grown on a LB agar plate, and a single colony was selected from each plate and inoculated into 10 mL of sterile LB broth overnight at 37 °C with shaking at 250 rpm. Thereafter, each culture was diluted to OD_600_ = 0.165 (approximately 1.5 ×10^8^ CFU/mL), and subsequently to 1:5000. 10 µL of the 1:5000 bacterial culture was added to the wells of a 96-well plate along with 90 µL of LB broth. Each recombinant peptide was added to the wells containing bacterial colonies to a final concentration of 100 µg/mL in a final well volume of 200 µL. 100 µg/mL of pexiganan (for *E. coli*, *P. aeruginosa,* and *B. subtilis*; [44]) and 100 µg/mL vancomycin (for *S. aureus;* [45]) were included as positive controls. Negative controls consisted of 10 µL of the 1:5000 bacteria culture and 100 µL PBS (pH 7.4). Each experiment was conducted in triplicate. The 96-well plate was incubated at 37 °C with shaking at 250 rpm for 210 min. The Miles and Misra colony counting method was used to measure antibacterial activity, as previously described [24], [46]. After incubation, a 10 µL aliquot was retrieved from each active well, diluted 1:10, 1:100 and 1:1000 using sterile filtered PBS, plated onto LB agar plates, allowed to dry completely at 25 °C, and finally incubated at 37 °C for 18 h. For each of the three replicates included for each condition, colony counts were performed from the LB agar plates containing the least diluted bacteria that yielded distinct colonies. The number of bacterial colonies formed on negative control plates was also counted. The following formula was applied to calculate the number of colony-forming units (CFU)/mL in each replicate: CFU/mL = (number of colonies × 20 × dilution factor) x 5. GraphPad Prism version 5.01 was used to compare the CFU/mL values in the presence of recombinant peptide with the CFU/mL values of the negative control, and bactericidal activity was determined using one-way analysis of variance (ANOVA) with Tukey’s multiple comparison test. A reduction in CFU/mL values at *P* ≤ 0.05 was considered statistically significant.

### 2.6 Microfluidics device fabrication and cell loading

Fabrication of the mother-machine microfluidic platform was achieved using polydimethylsiloxane (PDMS) base elastomer and curing agent, which were combined and mixed at a ratio of 10:1. To eliminate air bubbles, the mixture underwent a degassing process in a desiccator for 30 min. The degassed mixture was uniformly spread over a silicon wafer, which had microfluidic design features pre-fabricated using laser lithography. A secondary degassing step was implemented to ensure the removal of any remaining bubbles. The PDMS was baked at 95 °C for 1 h. Individual mother-machine microfluidic chips were cut from the PDMS mould, and holes for inlets and outlets were created using a 0.75 mm biopsy puncher. Subsequently, the PDMS chips and coverslips were cleaned. The PDMS chips were submerged in isopropanol and sonicated for 30 min, then dried using an air gun and baked at 95 °C for 30 min. The PDMS chips were sonicated again in deionized (DI) water for 30 min. The coverslips were first sonicated in 1 M potassium hydroxide for 20 min, and then in DI water for another 20 min. The clean coverslips were dried with an air gun and incubated for 30 min at 95 °C. 24 h prior to the experiments, the feature side of the cleaned PDMS chip was bonded to a clean coverslip using plasma treatment (2 min at 35 W and air pressure set to 0.2–0.3 mbar). Post-bonding, the devices were baked for 1 h at 95 °C.

Immediately before the experiments, the devices were passivated and cleaned by flowing LB + pluronic (0.8 % of a 0.1 g/mL stock) through the microfluidic lanes using a pair of gel-loading tips. To load the cells into the microfluidic device, 1 mL of overnight bacterial culture was concentrated by centrifugation at 1000 x g for 3 min and then resuspended in 50 µL LB medium. A small amount of resuspended high-density cells was aspirated with special gel-loading pipette tips and loaded into the lanes of the mother-machine devices through the inlets. The loaded chips were centrifuged at 1000 x g for 1 min to place the cells into the narrow cell-trenches orthogonal to the flow lane. Subsequently, fresh LB was flown through the device to clear the feeding lane and provide nutrients to the cells in the trenches.

### 2.7 Timelapse microscopy of antimicrobial effects on cells

Timelapse microscopy and effects of selected recombinantly expressed helminth AMPs on selected bacteria was achieved using a Nikon ECLIPSE Ti2 with a 40 × 0.95NA objective lens and a 1.5x post-objective magnification. The images were acquired using a Hamamatsu ORCA-Fusion Digital CMOS camera with a pixel size of 6.5 µm x 6.5 µm. Samples were illuminated with a brightfield light source using the phase-contrast setup. The mother-machine was fixed onto the stage inside an incubator maintained at 37 °C with a continuous supply of fresh autoclaved LB media and LB media containing individual recombinant AMPs through silicone tubing. The outlet was connected to a waste bottle. Focal drifts from minor thermal fluctuations were eliminated by the Nikon Perfect Focus System (PFS), and data acquisition was controlled by the Nikon elements software. This allowed time-lapse imaging of multiple fields of view (FOVs). The cells were imaged using phase contrast mode every 1 min with 100 millisecond exposure per frame, and PFS turned on throughout the experiment.

### 2.8 Image processing and single-cell data analysis

Image data were saved in ND2 format and subsequently converted to individual TIFF snapshots for each FOV using custom Python scripts. The images were registered in time-series to compensate for any stage drift caused by minor thermal fluctuations, then extracted into single-trench images using custom-designed image-processing pipelines, as described in earlier work [47]. Cells were detected and outlined from the single-trench images using the deep-learning image-segmentation algorithm Omnipose [48], which was retrained with synthetic images of cells in microfluidic trenches generated using the virtual microscopy platform SyMBac [47]. A custom-designed lineage tracking program (https://github.com/erezli/MMLineageTracking) was used to track the cell lineages over time. Cell size timeseries data were extracted from each tracked lineage and growth rate of individual cells and lysis frequency were computed from these data.

## 3 Results

### 3.1 Literature search and in silico AMP predictions

A total of 3870 accession numbers were retrieved from 12 publications describing proteomics datasets of ESPs from eight GI parasitic nematodes, i.e. *A. suum, H. contortus, Heligmosomoides polygyrus, Nippostrongylus brasiliensis, Strongyloides ratti, T. circumcincta, Toxocara canis,* and *Trichuris suis* (not shown). Of these, 3749 were successfully matched with their respective protein sequences (Table S1). A search for proteomics datasets generated from EVs of GI parasitic nematodes yielded a total of 850 accession numbers from *A. suum, H. polygyrus, N. brasiliensis,* and *T. circumcincta*, all of which were successfully matched with their respective protein sequences (Table S1).

Complete lists of ESP- and EV-associated protein sequences and corresponding GO and InterPro annotations, together with putative antimicrobial activity scores (based on CAMP_R3_ BLASTp, ampir and MultiPep analyses, see Methods) are provided in Table S2. Of the 3749 ESP-associated protein sequences subjected to downstream analyses, three were selected for further characterisation based on high antimicrobial activity prediction scores, i.e., a metridin ShK toxin domain-containing peptide from *H. contortus* (*H*met), a histone H2A from *T. canis* (*Tc*his), and a destabilase from *T. circumcincta* (*T*des) (Table S2). *H*met is composed of 84 amino acid residues with a signal peptide from residue 1–20. The protein includes a ShK domain-like region (residues 45 to 84) and a region with membrane-binding function (residues 24 to 84) (Table S2). No significant similarity between *H*met and known AMPs in the CAMP_R3_ database was identified. Nevertheless, *H*met was classified as a putative AMP by both ampir (score = 0.996) and MultiPep (0.746) (Table 1). *Tc*his (126 amino acid residues) displayed significant sequence similarity to a known antibacterial histone H2A derived from the whiteleg shrimp *Litopenaeus vannamei* (E-value: 4E^-75^) and was classified as a putative AMP by ampir (0.992) and MultiPep (0.958) (Table 1; Table S2). *T*des (139 amino acid residues) belongs to the invertebrate-type lysozyme protein family and shares significant similarity to a destabilase from the Eastern oyster, *Crassostrea virginica* (E-value: 4E^-39^). This protein contains a N-terminal signal peptide and a membrane-binding region that includes the destabilase/lysozyme domains (residues 18 to 139) (Table S2). *T*des was assigned an ampir score of 0.923 and a MultiPep score of 0.440 (Table 1).

**Table 1:** Top-scoring putative antimicrobial peptides identified in available proteomics datasets from excretory-secretory products (ESPs) and extracellular vesicles (EVs) from gastrointestinal helminth species. Putative antimicrobial activity was assigned based on comparative analyses with sequence data available from the Collection of Anti-Microbial Peptides (CAMP_R3_) database, as well as according to antimicrobial protein and peptide (AMP) prediction algorithms (ampir and MultiPep). For each sequence, helminth species of origin, corresponding Uniprot accession number, NCBI/Uniprot protein description and top CAMP BLAST hit are provided, as well as ampir and MultiPep antimicrobial/antibacterial prediction scores.

Sequence_ID	Species	Accession	Description	CAMP BLAST hit	ampir	MultiPep – antimicrobial	MultiPep – antibacterial
*H*met	*Haemonchus contortus*	A0A7I4Y4R3	Metridin-ShK toxin	NA	0.996	0.746	0.215
*Tc*his	*Toxocara canis*	A0A0B2V2B5	Histone 2A	Histone H2A	0.992	0.325	0.958
*T*des	*Teladorsagia circumcincta*	A0A2G9TVU4	Destabilase (Lysozyme)	Lysozyme 3	0.923	0.440	0.049
*T*sap	*Teladorsagia circumcincta*	transcript/25448 (iso-seq)	Saposin-b	Caenopore-5	0.864	0.943	0.279
*T*scp	*Teladorsagia circumcincta*	A0A2G9TU46	SCP-like protein	NA	0.732	0.003	0.000

Targeted analyses of 850 EV-associated sequences yielded two proteins of interest, both originating from *T. circumcincta*, i.e., a cysteine-rich secretory protein (*T*scp) and a saposin-b domain containing peptide (*T*sap). *T*scp (166 amino acid residues) contains a predicted signal peptide (residues 1 to 18) and a membrane-binding region (19 to 166), including a ‘*C*ysteine-rich secretory proteins, *A*ntigen 5, and *P*athogenesis-related 1 proteins’ (CAP) domain (31 to 166) (Table S2). *T*scp was assigned an ampir score of 0.732 (Table 1). *T*sap (104 amino acids) shared significant sequence similarity to a known AMP from the free-living nematode *Caenorhabditis elegans* (i.e., caenopore-5; E-value: 2E^-9^) (Table S2). *T*sap was assigned ampir and MultiPep scores of 0.864 and 0.942, respectively (Table 1).

### 3.2 Peptide structure prediction and characterisation

**Fig. 1:**
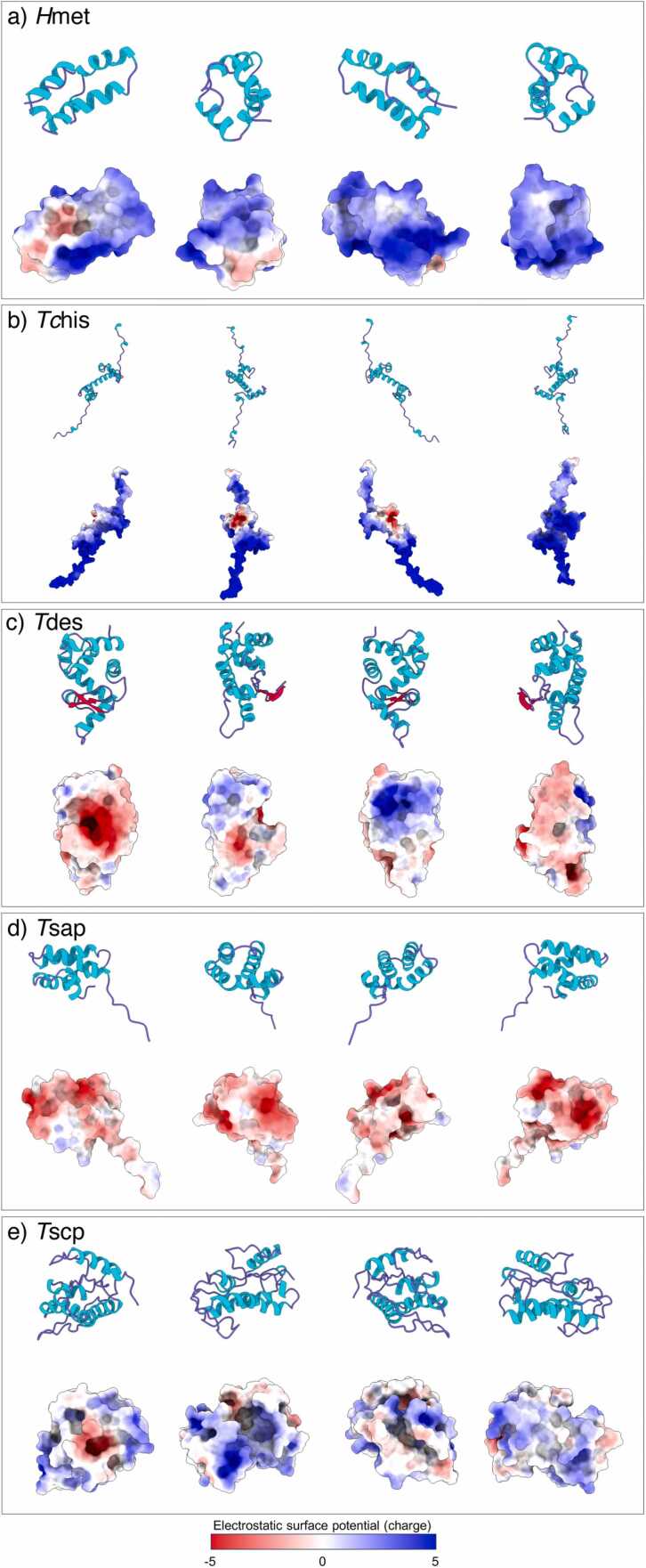
Predicted tertiary structures and electrostatic surfaces of selected nematode-derived antimicrobial peptides. (a) *Haemonchus contortus* metridin ShK toxin domain-containing peptide (*H*met), (b) *Toxocara canis* histone H2A (*Tc*his), (c) *Teladorsagia circumcincta* destabilase (*T*des), (d) *T. circumcincta* saposin-b domain containing peptide (*T*sap), and (e) *T. circumcincta* cysteine- rich protein (*T*scp). Robetta tertiary structures were predicted using RoseTTAFold and represented as ribbon structures (top). For each structure, predicted electrostatic surfaces were calculated using the APBS software at pH 7.0 (bottom). Red and blue indicate negative and positive charge, respectively. Each predicted structure is shown as four images, each representing a 90° turn. Visualisation was achieved using ChimeraX.

The tertiary structures of *H*met, *Tc*his, *T*des, *T*sap and *T*scp were predicted using Robetta RoseTTAFold, with corresponding confidence scores of 0.65, 0.77, 0.89, 0.87 and 0.76, respectively. Each model exhibits low predicted B-factor values, indicative of well-defined structures (Fig. S1). The *H*met model contains three disulphide bonds within the second and third α-helices and the C-terminus, respectively (Fig. S2a) and an overall positive charge distribution [+ 3.6 at pH 7.4 (pI = 8.5)] (Fig. 1a). The *H*met homodimer was predicted with high confidence, showing the formation of multiple predicted pseudobonds, with low PAE values, between each chain (Fig. S3). The *Tc*his model lacks disulphide bonds and is dominated by a net positive charge [+ 13.3 at pH 7.4 (pI = 10.6)], primarily distributed across the N- and C-terminal loops and on the two α-helices that flank the central 25-amino acid long α-helix (Fig. 1b). Several high confidence predicted pseudobonds were identified between *Tc*his chains during assessment of homodimer formation (Fig. S3). The *T*des model contains seven disulphide bonds (Fig. S2b) and a net negative charge [− 3.1 at pH 7.4 (pI = 6.3)]; however, electrostatic surface potential analysis predicts that the opposing faces of *T*des may be oppositely charged (Fig. 1c). *T*des homodimer prediction was associated with pseudobonds with low confidence (i.e., >10 Å) and therefore considered unlikely to form homodimers (Fig. S3). The *T*sap model contains three disulphide bonds between C13 – C87, C16 – C81, and C42 – C56 (Supplementary Fig. S2c), and a net charge of − 4.1 at pH 7.4 (pI = 5.3); electrostatic surface potential analysis (Fig. 1d), suggests that the surface of *T*sap may be dominated by a net negative charge. Few pseudobonds with high confidence were predicted to occur between two *T*sap chains (Fig. S5). The *T*scp model contains one disulphide bond (Fig. S2d) and is characterised by a predominantly positively charged surface [+ 0.98, pH 7.4, pI = 7.8] (Fig. 1e). *T*scp is unlikely to form homodimers, as suggested by high PAE values associated with the predicted pseudobonds within the homodimer complex (Fig. S3). To simulate potential interactions between candidate AMPs and the bacterial membrane, the association and arrangement between each model and Gram-negative/positive lipid bilayers were predicted using the PPM 3.0 server (https://opm.phar.umich.edu/ppm_server3). All peptide models interacted with and became embedded in the bacterial membrane, albeit no membrane penetration was predicted (Fig. S4).

### 3.3 Peptide expression and antimicrobial assays

**Fig. 2:**
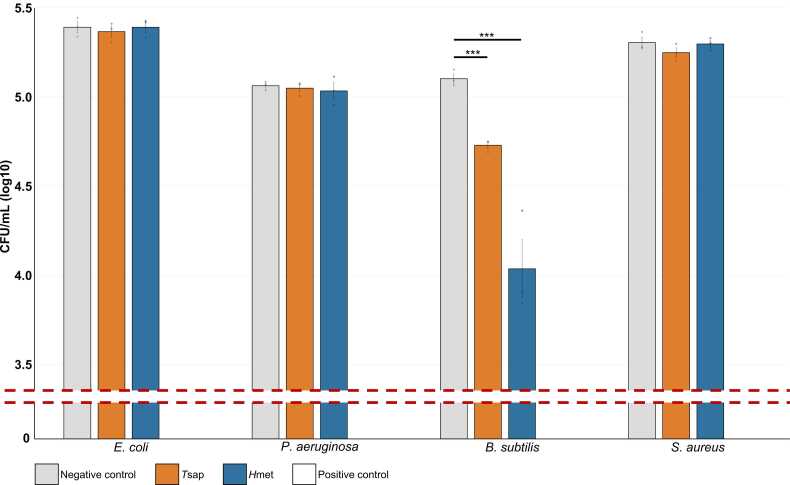
Bacterial growth assays. *Escherichia coli*, *Pseudomonas aeruginosa, Bacillus subtilis*, and *Staphylococcus aureus* were cultured in the presence of *Teladorsagia circumcincta* saposin-b domain containing peptide (*T*sap) and *Haemonchus contortus* metridin ShK toxin domain-containing peptide (*H*met) (100 μg/mL), as well as of negative (i.e., PBS) and positive controls [i.e., pexiganan (for *E. coli, P. aeruginosa*, and *B. subtilis*) and vancomycin (for *S. aureus*)] for 210 min. Following incubation, colony counts were performed and used to calculate the total CFU/mL in the respective cultures. Note that exposure to pexiganan and vancomycin resulted in complete elimination of bacterial colonies. Data are representative of four independent experiments. Individual datapoints reflecting biological replicates for each experiment are represented as coloured dots and error bars represent standard error of the mean. * ** *P* ≤ 0.001.

While all five ‘peptides of interest’ were selected for recombinant expression, two (i.e., *H*met and *T*sap), were successfully expressed in *K. phaffii* using standard expression conditions (Fig. S5a). Western blot analysis using monoclonal anti-histidine antibodies confirmed the presence of the poly-histidine tag on both *H*met and *T*sap. These were therefore purified using Ni-NTA agarose (Fig. S5b). Final concentrations of recombinant *H*met and *T*sap were 0.45 mg/mL and 0.62 mg/mL, respectively. At 100 μg/mL, neither *H*met nor *T*sap exerted a significant inhibitory effect on the growth of *E. coli*, *P. aeruginosa,* or *S. aureus* (Fig. 2). Conversely, exposure of *B. subtilis* to 100 μg/mL of either *H*met and *T*sap resulted in significant bacterial growth reductions, i.e., 90.0 % and 57.7 % CFU/mL respectively, when compared to negative controls (Fig. 2).

### 3.4 Antimicrobial activity of *H*met and *T*sap revealed by microfluidics time-lapse microscopy

**Fig. 3:**
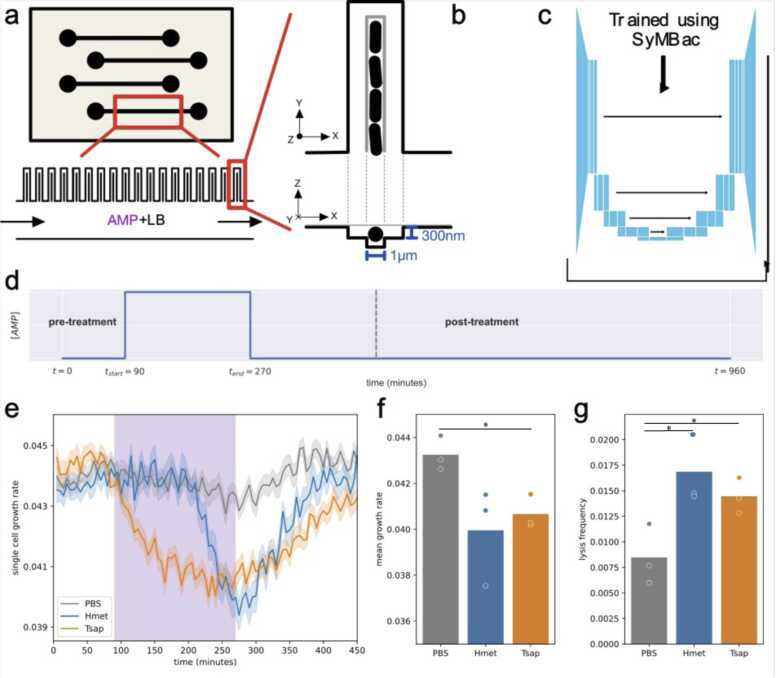
Time-lapse microfluidics assay. (a) Schematic diagram illustrating the modified Mother Machine device featuring side trenches for facilitating diffusive reach of *Haemonchus contortus* metridin ShK toxin domain-containing peptide (*H*met) and *Teladorsagia circumcincta* saposin-b domain containing peptide (*T*sap). The inset at the bottom demonstrates the placement of cell trenches alongside the flow lane, while the inset on the right provides a magnified view of the structure of individual cell trenches. (b) Example phase-contrast image depicting cells within a trench (1 µm wide), with visible side trenches (7 µm wide) shown in light grey. (c) Image segmentation of cells in phase-contrast using a machine-learning algorithm (Omnipose) trained with synthetic training data generated by the virtual microscopy platform SyMBac. (d) Schematic experimental timeline: growth media containing each *H*met and *T*sap were introduced after 90 min of pre-treatment with fresh LB, followed by a return to growth media (LB) without AMPs after 180 min of treatment. (e) Average single-cell growth rates under different treatments, as well as control, with the treatment interval highlighted in purple. Highlighted regions around each line represent the standard deviation for their respective treatment conditions. (f) Average growth rates measured between 250 and 300 min from three replicates, with individual values depicted as circles. (g) Average and individual lysis frequencies of bacterial lineages during treatment, as observed in three experiments. * *P* ≤ 0.05.

The effect of *H*met and *T*sap exposure on the physiology of individual *B. subtilis* cells was observed using time-resolved imaging of cells loaded in microfluidic devices. The ‘mother-machine’ microfluidic device design was selected as it enables the tracking of individual cell lineages under consistent growth conditions for extended time durations [49], [50]. These devices consist of fluidic lanes (Fig. 3a) with designated inlets and outlets for the continuous flow of growth media and treatment solutions. Five-hundred cell trenches, each measuring 75 µm in length and 1 µm in width, were positioned perpendicular to the flow lanes, such that a single line of cells occupied each trench. The trenches were constructed as dead-ends, ensuring that the cells at the dead-end remained in place throughout the experiment, while their offspring were removed from the open end (Fig. 3a). This setup allows cell lineages to be tracked within an environment that remains constant over time and is consistent across all trenches, ensuring reliable comparison of cell lysis events across trenches and over time. Additionally, the shallow side trenches adjacent to each main trench facilitate the diffusion of media and exposure of target cells to putative AMPs [51]. Phase-contrast imaging was used to acquire movies of single-cell growth dynamics before, during and after treatment (see Fig. S6 and Fig. S7 for *H*met and *T*sap, respectively). A representative image of cells within a trench is shown in Fig. 3b. We used a deep-learning image segmentation model (Omnipose [48]) trained with synthetic data generated from the virtual microscopy platform SyMBac [47] to identify and outline individual cells from the image data. A custom-designed lineage-tracking algorithm (https://github.com/erezli/MMLineageTracking) was used to track cells across frames and cell size timeseries were extracted from these tracks to quantify growth and lysis rates of individual cells (Fig. S8 and Fig. S9, respectively).

To assess the effect of *H*met and *T*sap exposure on *B. subtilis* cell physiology, a comparison of cell lysis rates and growth rates in two distinct fluidic lanes (treatment and control) was conducted. In the first lane, individual recombinant AMPs were administered for a duration of three hours, while cells in the other fluidic lane were treated with LB mixed with 50 % PBS. An illustrative timeline of this experiment is provided in Fig. 3d. We observed that both *H*met and *T*sap caused up to 10–15 % decrease in cell growth rates during the 3-h treatment. The effect of *H*met was stronger, and the results were consistent between replicates (Fig. 3f). Comparisons of the number of cell lysis events between the two flow lanes revealed a distinct pattern; in particular, exposure of *B. subtilis* to *H*met was associated with a significantly increased frequency of cell lysis following treatment (Fig. 3g). However, the observed lysis frequencies estimated from this single-cell data were too low to account for the results from the bulk colony count treatment data. We developed a simple computational model to assess the population-level impacts of growth inhibition and lysis rate (Fig. S10) and found that the effect of growth inhibition explains the decrease in colony counts observed in the bulk experiments.

## 4 Discussion

In this study, we mined available proteomics data from ESPs and EVs of GI nematodes of substantial veterinary and public health importance to identify and characterise putative AMPs, and applied in vitro techniques to study one of the potential mechanisms governing worm-microbiota interactions in the vertebrate GI tract. Using predictive algorithms, we shortlisted five putative AMPs from the ‘brown stomach worm’, *T. circumcincta* (i.e., *T*sap, *T*des, and *T*scp), the ‘barber’s pole worm’, *H. contortus* (i.e., *H*met), and the zoonotic canine roundworm *T. canis* (i.e., *Tc*his), two of which (i.e., *T*sap and *H*met) were successfully expressed in recombinant form and shown to exert significant antibacterial activity against *B. subtilis*.

*T*sap belongs to a diverse family of saposin-like proteins (SAPLIPs), each sharing a conserved core structure, the ‘saposin fold’. This fold is a characteristic bundle of four or five α-helices, stabilised through the formation of three conserved disulphide bonds, that is central to the activity of these molecules [52], [53]. Variations in the amino acid sequences of SAPLIPs confer these proteins diverse functions, including but not limited to, roles in the activation of sphingolipid hydrolases, lipid antigen presentation, and processing of apoptotic bodies [54]. Of note, SAPLIPs have been shown to act as potent antimicrobials, as demonstrated by the ability of amoebapores from *Entamoeba histolytica,* human granulysin, and porcine NK-lysin to disrupt the outer membranes of parasites, bacteria, and fungi [54]*. T*sap shares significant sequence identity to a caenopore from *C. elegans*. Caenopores are known antimicrobials, exerting significant bactericidal activity against both Gram-positive and Gram-negative bacteria, such as *Bacillus megaterium* (caenopore-1, −5 and −12), *Bacillus thuringiensis* (caenopore-12), and *E. coli* (caenopore-5) [53]. *H*met contains four α-helices stabilised through the formation of three conserved disulphide bonds. Although no antimicrobial activity has thus far been documented for parasitic nematode-derived metridin ShK toxin domain-containing proteins, a study by O’Rourke *et al.* [55] reported that, in *C. elegans*, four metridin-like ShK toxin domain-containing proteins were expressed in response to infection by *Microbacterium nematophilum*. In a similar study, *C. elegans* genes encoding ShK toxins were upregulated in response to *P. aeruginosa* infection [56]. In addition, a known ShK toxin domain-containing AMP from the mesoglea of the jellyfish, *Aurelia aurita*, i.e., aurelin, was shown to exert antibacterial activity against *Listeria monocytogenes* and *E. coli* [57]*.*

While recombinant *T*sap and *H*met exerted significant antibacterial activity against *B. subtilis*, no effect was observed against *E. coli*, *P. aeruginosa,* or *S. aureus*. This observation may be linked to inherent differences between the membrane structures of each of these bacterial strains. Indeed, the killing of Gram-negative bacteria involves the disruption of both outer and cytoplasmic membrane, and the inability to permeabilize or disrupt the outer membrane results in loss of antimicrobial activity [58]. For Gram-positive bacteria, differences between the membrane composition of *B. subtilis* and *S. aureus* may be responsible for the observed discrepancy between killing activities. Indeed, while the membrane of *B. subtilis* contains glycans with an average strand length of 500 disaccharide units, *S. aureus* glycans are short (∼5 −10 disaccharide units) and feature an extremely high degree of peptide cross-links that confer substantial rigidity to the membrane of this bacterium [59]. Additional studies examining the antimicrobial properties of nematode-derived AMPs against an expanded panel of bacterial targets are necessary to clarify whether any bactericidal/bacteriostatic activity is dependent upon bacterial membrane structure. For example, the membrane of *Streptococcus pneumoniae* features glycans with an average length of 50 subunits, thus making it a potentially useful intermediary between *B. subtilis* and *S. aureus* [60].

While the exact antibacterial mechanism of action cannot be determined for either *H*met nor *T*sap, *B. subtilis* exposure to each AMP was associated with statistically increased frequency of cell lysis during microfluidics time-lapse microscopy. Nevertheless, these lysis frequencies were too low to account for the substantial reduction of *B. subtilis* CFUs during the colony counting experiments. Instead, analysis of simulated *B. subtilis* cultures, using the lysis frequencies and growth rates observed during the microfluidics experiments, suggested that such diminished growth rates are likely responsible for the significantly reduced *B. subtilis* CFUs recorded during the colony counting experiments. However, it is important to note that the effects observed in the single-cell microfluidic assay are likely to be weaker than those in the bulk colony counting assay due to inherent differences between experimental setups. Indeed, during the microfluidics experiment, *H*met and *T*sap are likely to preferentially bind to cells near the trench inlet and thus quickly become depleted, as evidenced by the decrease in growth inhibition effect along the trench; conversely, in the colony counting assay, whole bacterial colonies are exposed to the presence of the AMPs in equal measure. Nevertheless, we are tempted to speculate that *H*met and *T*sap exert their antibacterial activity via interaction with and disruption of the bacterial membrane, as evidenced by the increased lysis events observed during treatment. In the future, techniques such as atomic force microscopy and/or electrochemical scanning tunnelling microscopy could be applied to clarify the mechanism of action of these AMPs [61], as testified by the application of such techniques to visualise and characterise the mechanism of bacterial membrane disruption caused by alamethicin, a channel-forming peptide [61].

In our previous study, we showed that adult *T. circumcincta* ESPs exert significant antibacterial activity against *E. coli* [24]*,* a finding that could not be replicated in our current investigation on *H*met and *T*sap. While it is plausible that these AMPs may not be active against *E. coli*, it is also possible that this discrepancy may be linked to substantial differences between in vitro and ‘real-world’ conditions. For instance, the abomasa of sheep and goats naturally infected by *T. circumcincta* and *H. contortus* often harbour tens of thousands of worms, each continuously producing antimicrobial-containing ESPs and EVs [62], [63]. Thus, the hypothesis that *T*sap and/or *H*met may exert antimicrobial activity at higher concentrations than those tested in our study, and/or act synergistically with other nematode ESP and/or EV components to enact bactericidal activity against selected bacterial species (e.g., *E. coli*), cannot be excluded. In addition, it is worth noting that optimum caenopore activity is observed under acidic pH conditions (= 5.2; [64]). Interestingly, the pH of the abomasum of small ruminants, in which both *T. circumcincta* and *H. contortus* reside, increases substantially during GI helminth infection (i.e., from ∼2.9 to 4.5 – 5.5) [13], [65]. Thus, future investigations should focus on adapting experimental parameters to closely resemble the conditions occurring over the course of natural infections by these nematodes. It should also be noted that one or more of the remaining shortlisted peptides that we were unable to express in the current study (i.e., *Tc*his, *T*scp, and *T*des) could be active against *E. coli,* and/or *P. aeruginosa,* and/or *S. aureus*. Indeed, histone H2A from *Oncorhynchus mykiss* has been shown to possess potent antibacterial activity against *Aerococcus äiridans*, *B. subtilis,* and *Micrococcus luteus* [66], while human histone H4 exerts antimicrobial activity against *S. aureus* and *Propionibacterium acnes* [67]. In *C. elegans*, genes encoding SCPs have also been shown to be upregulated during infections by several bacterial pathogens [55]*.* Finally, destabilases exert antibacterial activity by enzymatically cleaving a glycosidic linkage within the peptidoglycan of bacterial cell walls [68]. Further optimisation of experimental conditions, such as culture temperature, methanol concentration, and vector feeding strategy, is required to achieve expression of *Tc*his, *T*scp, and *T*des [69]*.*

Our data support the notion that GI helminth ESPs and secreted EVs may communicate with and modulate the composition of their microbial surroundings [70]. In our previous study, *T. circumcincta* infection was associated with significant changes in the ovine GI microbiota, and it was observed that increased abundances of Bacilli, including bacteria from the genera *Turicibacter, Izemoplasmatales* and the XIII AD3011 group, were strongly associated with the faecal microbiota of uninfected lambs rather than that of *T. circumcincta*-infected animals [70]. Nevertheless, infections were equally associated with abundance reductions and expansions of several other taxa less related to *B. subtilis* [70]*.* However, the antimicrobial activity of helminth ESPs/EVs likely represents only one of several (direct and indirect) mechanisms governing worm-microbiota interactions. Indeed, multiple other factors, including but not limited to, physical and biochemical changes in GI environment in response to parasite colonisation, nutrient availability, and host immune responses, are likely to greatly contribute to pathogen-microbiota crosstalk [20]. In addition, fundamental changes in the GI microbial ecosystem due to worm establishment may trigger a complex network of bacteria-bacteria interactions, via e.g., quorum sensing, biofilm formation, release of bacterial EVs, and dynamic competition for nutrients [71], that are difficult to investigate using reductionist systems such as the one applied in this study. Future studies may exploit holistic technologies to explore the effect of recombinant helminth AMPs on whole microbial ecosystems. For instance, in a recent experiment, the antimicrobial activity of EVs from the rumen fluke, *Calicophoron daubneyi*, was assessed in an in vitro rumen model consisting of rumen fluid and anaerobic incubation medium [72], [73]. Furthermore, the application of ovine epithelial organoids to studies of worm-microbiota crosstalk holds substantial promise for the identification of host components that may contribute to this complex network of interactions [74]. Of note, the transcriptional profiles of ovine abomasal and ileal organoids were demonstrated to largely resemble those of the respective native tissues and remained stable after at least five serial passages [74]. Crucially, both types of organoids were successfully challenged with and infected by *T. circumcincta* L3s and, separately, by *Salmonella enterica serovar* Typhimurium [74], thus providing a solid basis for co-culture experiments aimed to investigate helminth-microbiota interactions under controlled conditions.

A recent analysis of publicly available sequence data from a range of parasitic helminths identified over 16,000 putative AMPs encoded by the genomes of 127 worm species [75]. Building upon our findings and those by Irvine *et al*. [75], in depth structural and functional characterisations of helminth-derived AMPs may provide clarity on mechanisms of worm-microbiota interactions in vivo, and thus pave the way toward the development of novel and sustainable approaches to control parasitic diseases amid the ever-increasing threat of anthelmintic resistance. For instance, the rational manipulation of the ruminant gut microbiota (e.g. via the administration of pre- and/or probiotics and/or dietary supplements) may serve to counteract the effects that helminth AMPs exert on the overall gut microbial make-up, possibly enhancing host resistance to colonisation by GI helminths [76].

## 5 Conclusion

In this study, we investigated a yet untapped repertoire of AMPs in the ESPs and EVs of several GI helminths. Our experiments show that GI helminths may directly modulate their immediate microbial surroundings through the secretion of AMPs with cell lysis activity. However, experiments conducted in vivo or ex vivo (e.g. through exposure of whole gut inocula through one or multiple recombinant helminth AMPs) will be necessary in order to validate our hypothesis. Moreover, it is highly likely that worm-microbiota crosstalk relies on a complex network of direct and indirect mechanisms that act synergistically to enhance parasite survival in a hostile environment. Unravelling the complexities of helminth-host microbiome relationships may lead to a better understanding of helminth biology and to the discovery and development of novel and sustainable parasite control strategies.

## Funding

JR is the grateful recipient of a PhD scholarship by the Biotechnology and Biological Sciences Research Council (10.13039/501100000268BBSRC) of the United Kingdom. The CC laboratory is funded by grants by the BBSRC, Isaac Newton Trust and the University of Cambridge. AHL is supported by a grant from the European Research Council (10.13039/100010663ERC) under the European Union’s Horizon 2020 research and innovation program [850974] and a grant from the 10.13039/100008398Villum Foundation [00025302]. AJN, KMcl and DRGP receive funding from the Scottish Government, Rural and Environment Science and Analytical Services Division (10.13039/100011310RESAS). The research in SB laboratory is funded by the Engineering and Physical Sciences Research Council (10.13039/501100000266EPSRC) of the United Kingdom [award no. G114746] and Wellcome Trust [grant number RG89305]. AZ is a recipient of a Transition To Independence (TTI) fellowship from the School of Biological Sciences at the University of Cambridge and supported by funding from the Rosetrees Trust (grant number JS16/TTI2021\1), the Isaac Newton Trust (grant number 21.22(a)iii) and the School of Biological Sciences at the University of Cambridge. TPJ has received funding from the European Union's Horizon 2020 research and innovation program under the Marie Sklodowska-Curie grant agreement no. 713683 (COFUNDfellowsDTU).

## CRediT authorship contribution statement

**Somenath Bakshi:** Formal analysis, Methodology, Supervision, Writing – review & editing. **Ashraf Zarkan:** Methodology, Supervision, Writing – review & editing. **Cinzia Cantacessi:** Conceptualization, Funding acquisition, Project administration, Supervision, Writing – original draft, Writing – review & editing. **James Rooney:** Conceptualization, Data curation, Formal analysis, Methodology, Validation, Writing – original draft, Writing – review & editing. **Esperanza Rivera-de-Torre:** Methodology, Supervision, Writing – review & editing. **Ruizhe Li:** Data curation, Formal analysis, Methodology, Writing – review & editing. **Kevin Mclean:** Data curation, Writing – review & editing. **Daniel Price:** Data curation, Writing – review & editing. **Alasdair Nisbet:** Methodology, Writing – review & editing. **Andreas Laustsen:** Funding acquisition, Writing – review & editing. **Timothy Jenkins:** Methodology, Supervision, Writing – review & editing. **Andreas Hofmann:** Data curation, Formal analysis, Methodology, Writing – review & editing.

## Declaration of Competing Interest

The authors declare that they have no known competing financial interests or personal relationships that could have appeared to influence the work reported in this paper.
