# Supplementary material for: Structural and functional analyses of nematode-derived antimicrobial peptides support the occurrence of direct mechanisms of worm-microbiota interactions
Source: Comput Struct Biotechnol J. 2024 Apr 10;23:1522–33. doi: 10.1016/j.csbj.2024.04.019 (PMC11021794; doi:10.1016/j.csbj.2024.04.019)
Supplement: Supplementary file 1 — Supplementary material [file mmc1.docx]

**Supporting Information**

**Table S1. Available proteomics sequence data of gastrointestinal nematode excretory-secretory products (ESPs) and extracellular vesicles (EVs) at the time of writing.** Available ESP and EV proteomics datasets from selected gastrointestinal nematode species were retrieved and subjected to targeted bioinformatics analyses for the identification of putative antimicrobial proteins and peptides. For each species, life cycle stage and numbers of proteins/peptides described in the corresponding publications, as well as the numbers of proteins/peptides that could be successfully retrieved, are provided.

Abbreviations. L3 = third-stage larva; L4 = fourth-stage larva.

**Table S2. Putative antimicrobial proteins identified in available proteomics datasets from excretory-secretory products (ESPs) and extracellular vesicles (EVs) from gastrointestinal nematode species.** List of proteins retrieved from available proteomics analyses of gastrointestinal nematode ESPs/EVs. For each sequence, the following information is provided: SequenceID/Accession (Used in original source), species, gene ontology IDs, InterProScan IDs, top CAMP BLAST hit, and relevant information (Accession numer, Score, Evalue, Identities (%), Positives (%)), antimicrobial probability prediction scores (using the ampir ‘precursor’ and ‘mature’ classification models and MultiPep antimicrobial and antibacterial scores), and complete protein sequence.

**Fig. S1. Predicted structures of selected nematode-derived antimicrobial peptides.** (a) *Haemonchus contortus* metridin ShK toxin domain-containing peptide (*H*met), (b) *Toxocara canis* histone H2A (*Tc*his), (c) *Teladorsagia circumcincta* destabilase (*T*des), (d) *T. circumcincta* saposin-b domain containing peptide (*T*sap), and (e) *T. circumcincta* cysteine- rich protein (*T*scp). B-factor values (Å^2^) were generated per residue using the Robetta RoseTTAFold software and visualised using ChimeraX. Dark blue residues are indicative of low B-factor values and thus high accuracy of the model. Dark red residues are indicative of high B-factor values and thus low confidence scores. The range of B-factor values (Å^2^) calculated for each model, from lowest to highest, is provided under each predicted structure.

**Fig. S2. Predicted disulphide bonds within selected nematode-derived antimicrobial peptide models.** Predicted disulphide bonds within (a) *Haemonchus contortus* metridin ShK toxin domain-containing peptide (*H*met), (b) *Teladorsagia circumcincta* destabilase (*T*des), (c) *T. circumcincta* saposin-b domain containing peptide (*T*sap), and (d) *T. circumcincta* cysteine- rich protein (*T*scp). α-helices in cyan, β-sheets in red, loop regions in purple and cysteine residues in gold (with position labelled). Predicted disulphide bonds are indicated with yellow dashed lines. Disulphide bonds were predicted, measured, and visualised within ChimeraX.

**Fig. S3. Predicted formation of homodimers of selected nematode-derived antimicrobial peptides.** (a) *Haemonchus contortus* metridin ShK toxin domain-containing peptide (*H*met), (b) *Toxocara canis* histone H2A (*Tc*his), (c) *Teladorsagia circumcincta* destabilase (*T*des), (d) *T. circumcincta* saposin-b domain containing peptide (*T*sap), and (e) *T. circumcincta* cysteine- rich protein (*T*scp). Alphafold Colabfold was utilised to predict the likelihood of each peptide forming a homodimer. These predictions are visualised in ChimeraX in a globular form (left) which details the orientation of peptide chains interlocking in the homodimer complex, and as ribbons (right), where interactions between peptide chains are represented by ‘pseudobonds’. These are coloured according to their predicted alignment error (Å) values, ranging from 0 – 20 Å as indicated in the key. In both depictions, separate peptide chains of the predicted homodimer complex are represented in cyan and red, respectively.

**Fig. S4. Predicted orientation of selected nematode-derived antimicrobial peptides in a generalised bacterial lipid bilayer.** (a) *Haemonchus contortus* metridin ShK toxin domain-containing peptide (*H*met), (b) *Toxocara canis* histone H2A (*Tc*his), (c) *Teladorsagia circumcincta* destabilase (*T*des), (d) *T. circumcincta* saposin-b domain containing peptide (*T*sap), and (e) *T. circumcincta* cysteine- rich protein (*T*scp). Each predicted interaction between putative AMP and lipid bilayer is illustrated in four images, each representing a 90° turn. Peptides in navy blue, phosphate heads of the phospholipid within the lipid bilayer in red and fatty acids of the phospholipids in beige. The interaction between each protein model and a generalised bacterial lipid bilayer was calculated using the OPM PPM, rebuilt in CHARMM-GUI and visualised in ChimeraX.

**Fig. S5. Expression and purification of selected nematode-derived antimicrobial peptides.** (a) Expression of recombinant *T*sap (left) and *H*met (right) as detected in the supernatant of *Komagataella phaffii* (formerly known as *Pichia pastoris*) culture media. The purification of recombinant proteins from the supernatant was achieved using Ni-NTA agarose, which binds the N-terminal His-tag on the target protein. Each step of the purification process was visualised through SDS-PAGE. SN; Supernatant, FT; Flowthrough, W1; Wash 1, W2; Wash 2, E1; Elution 1, E2; Elution 2, E3; Elution 3, L; Molecular weight ladder. (b) Purified *H*met and *T*sap were probed for presence of the N-terminal His-tag using primary mouse anti-6His tag antibody, and secondary horse anti-mouse peroxidase antibody at a dilution of 1:250 and 1:400, respectively. The His-tag was detected in both purified protein products.

**Fig. S6**. Representative movie of single-cell growth dynamics before, during, and after exposure of *Bacillus subtilis* to *Haemonchus contortus* metridin ShK toxin domain-containing peptide (*H*met).

**Fig. S7**. Representative movie of single-cell growth dynamics before, during, and after exposure of *Bacillus subtilis* to *Teladorsagia circumcincta* saposin-b domain containing peptide (*T*sap).

**Fig. S8.** **Single-cell segmentation and lineage tracking.** (a) Phase-contrast images of cells were segmented using the machine-learning model Omnipose, trained with synthetic data from SyMBac. The segmentation process produces binary masks of individual cells (bottom left), that are ranked based on their positioning within each trench (with the mother cell darkest at the top). Subsequently, a custom-designed lineage tracking algorithm (<https://github.com/erezli/MMLineageTracking>) is employed to identify and label the tracks of individual cells accordingly (bottom right). Lineage connections between different cells are overlaid onto the raw phase-contrast image for illustrative purposes (top right).

**Fig. S9. Single cell growth and lysis analysis.** (a) A time series of individual cell lengths is estimated from their respective masks. The length of individual cells sharply decreases at the point of division and grows exponentially between division points. Consequently, the logarithm of cell length exhibits linear growth between division points, and its slope is utilised to calculate the elongation rate (exponential growth rate) of individual cells. Lysis events are identified when a lineage is abruptly truncated.

**Fig. S10.** (a) Population growth of four simulated cultures are compared for 210 minutes at different growth rates and lysis frequencies. The initial conditions (number of cells) and growth duration were set to match the bulk antimicrobial activity assays described in the main text. The growth rate and lysis frequencies were selected based on the single-cell AMP experiments. The impact of lysis appears to be significantly weaker than that of growth reduction in determining the final density of cells. (b) The final population cell counts after 210 minutes for different growth rates and lysis frequencies are compared. At reduced growth rates of 0.039 and 0.030 (s-1), a 2-fold and 8-fold reduction in cell count, respectively, are observed. In contrast, the frequency of lysis events has a less significant impact on the final cell count. (c) The antimicrobial peptides (AMPs) diffuse from the trench inlet on the right. (d) Growth rates are significantly reduced near the open end of the trench, likely because cells closer to the open end absorb more AMPs before the latter becomes depleted.
