# Supplementary figures and images for: Structural and functional analyses of nematode-derived antimicrobial peptides support the occurrence of direct mechanisms of worm-microbiota interactions
Source: Comput Struct Biotechnol J. 2024 Apr 10;23:1522–33. doi: 10.1016/j.csbj.2024.04.019 (PMC11021794; doi:10.1016/j.csbj.2024.04.019)

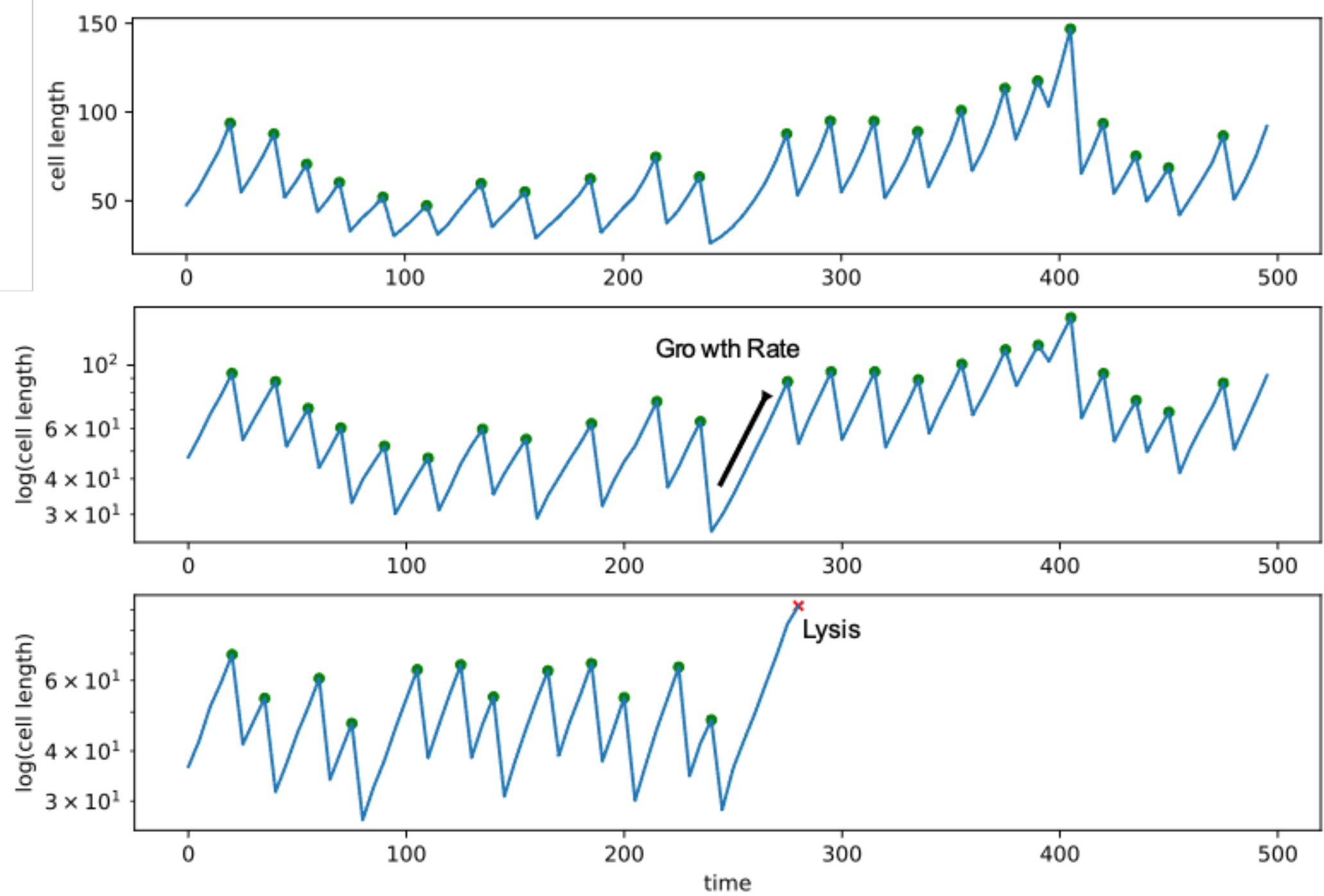

Supplement: Supplementary file 2 — Supplementary material [file mmc10.pdf]

**a**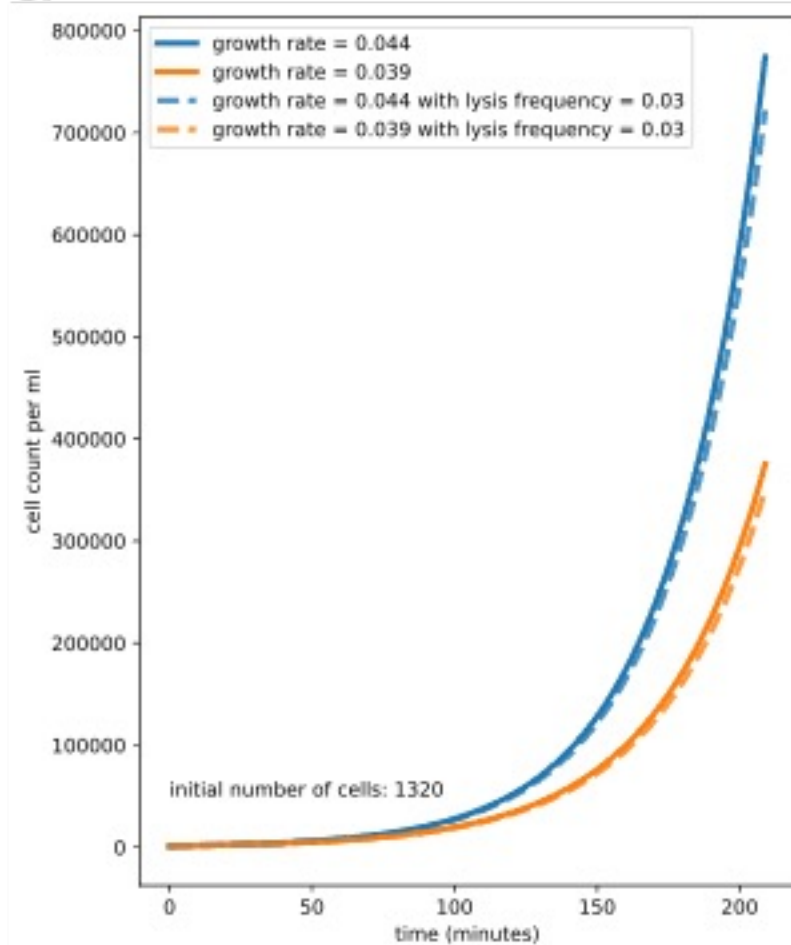**b**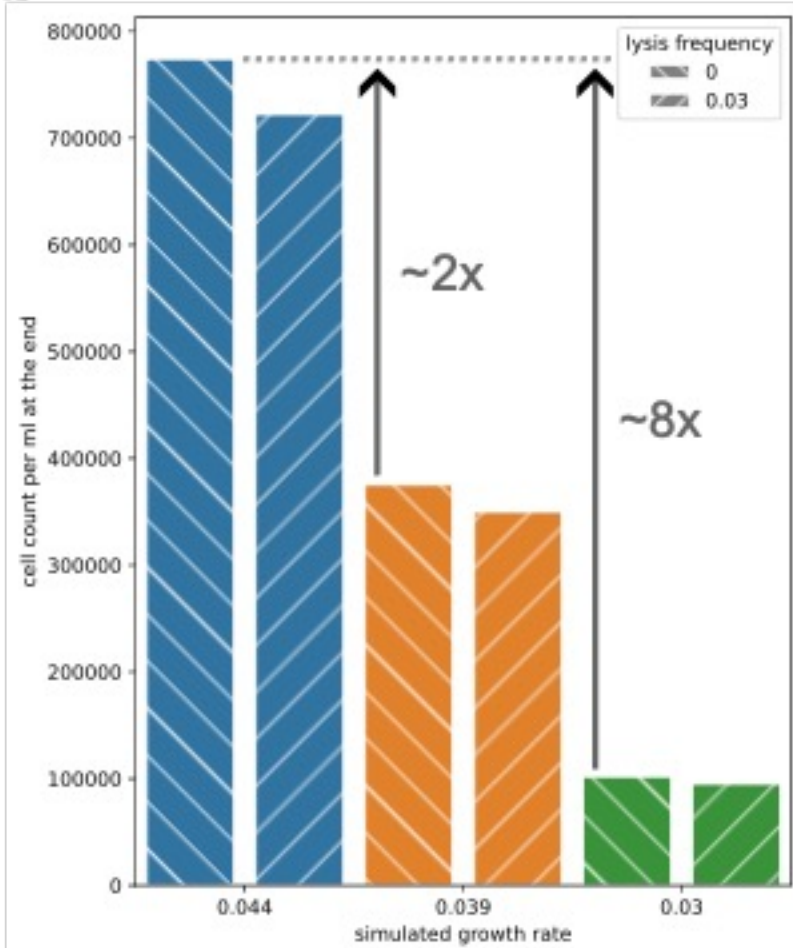**c**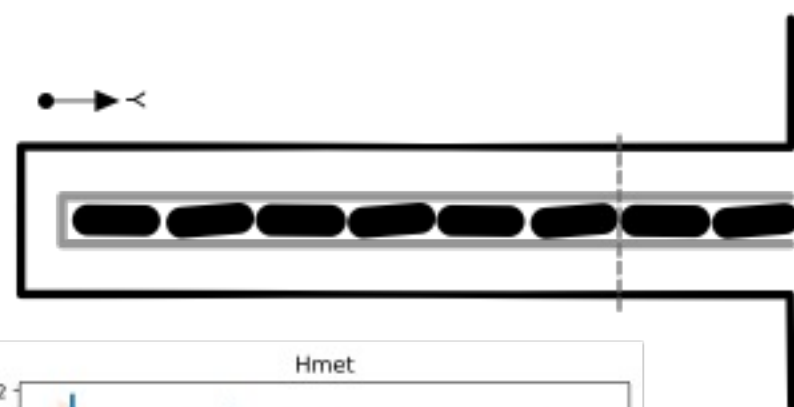**d**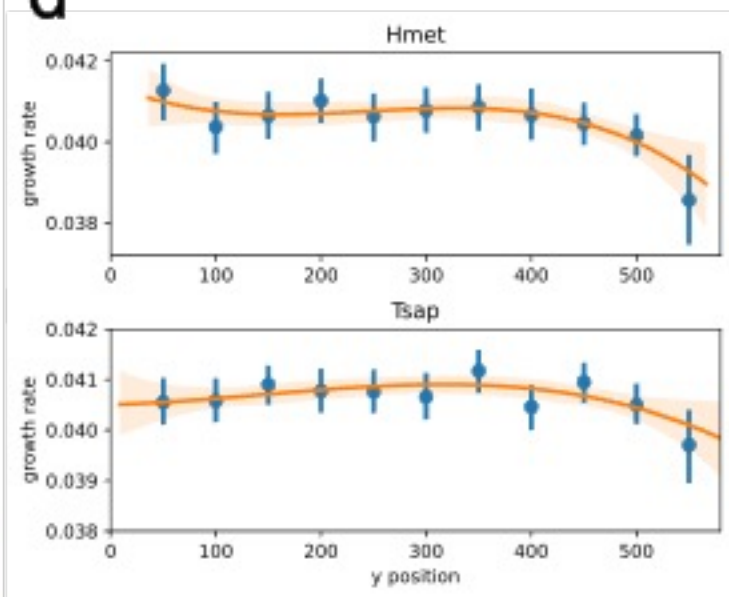

Supplement: Supplementary file 3 — Supplementary material [file mmc11.pdf]

a) *Hmet*

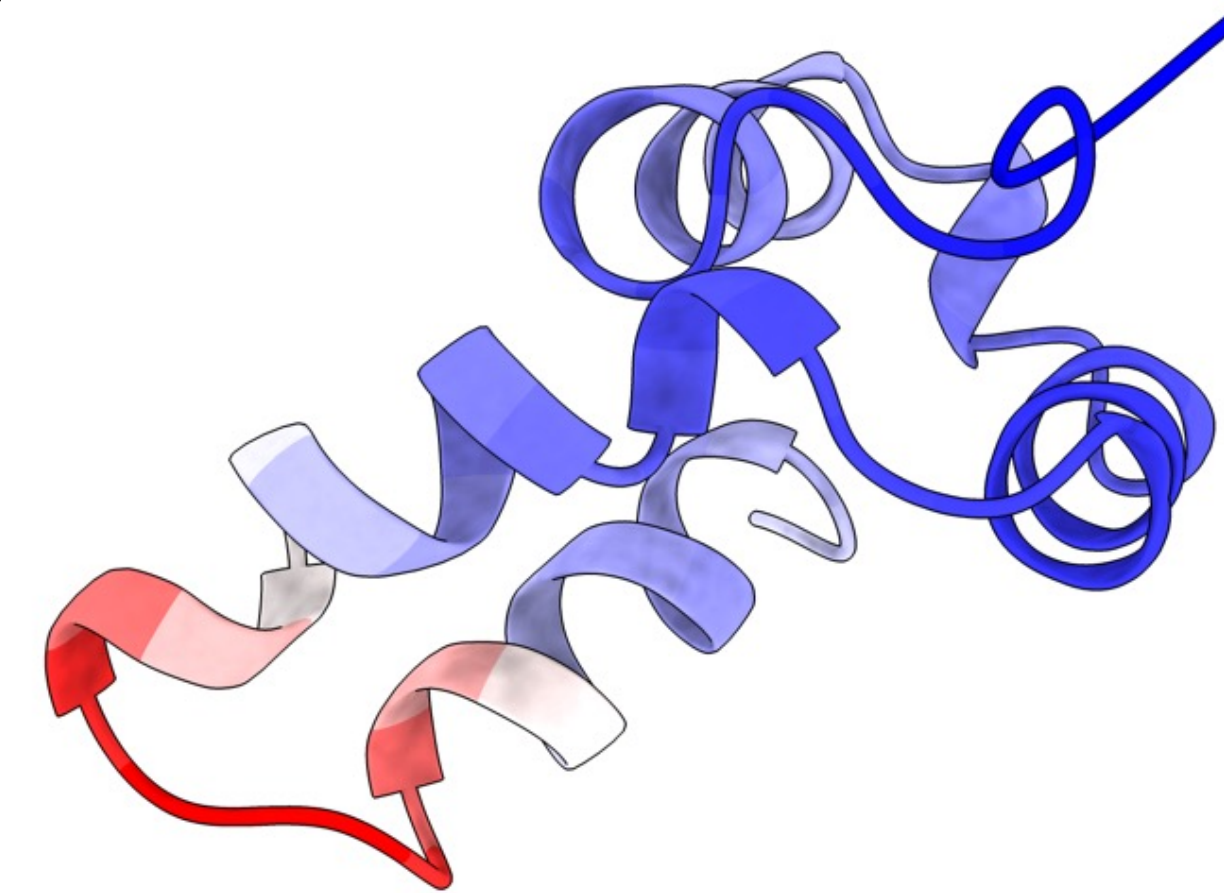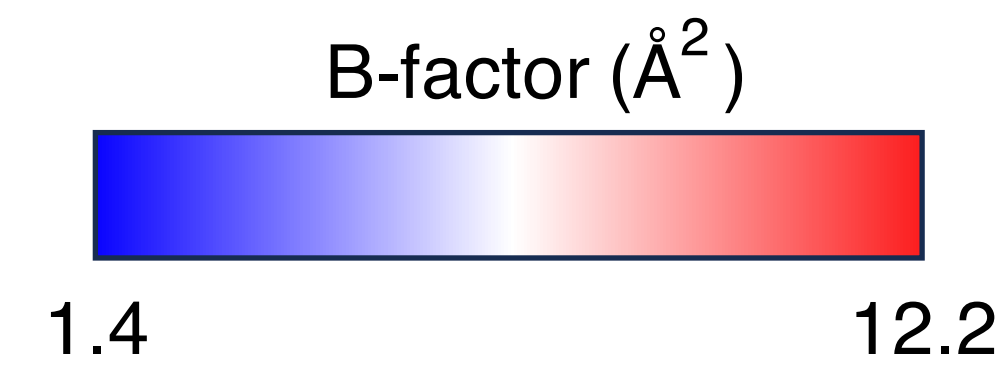

b) *Tchis*

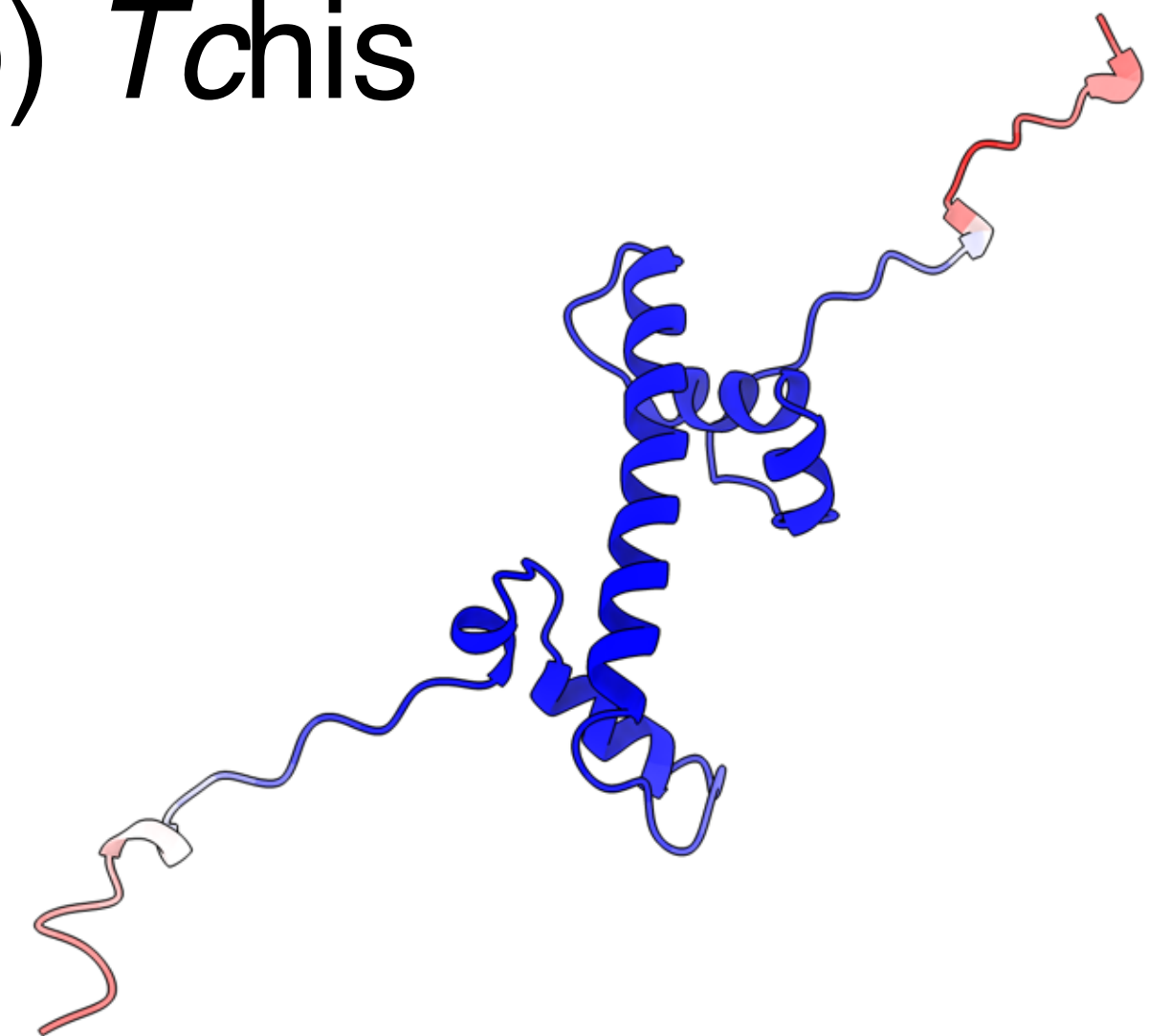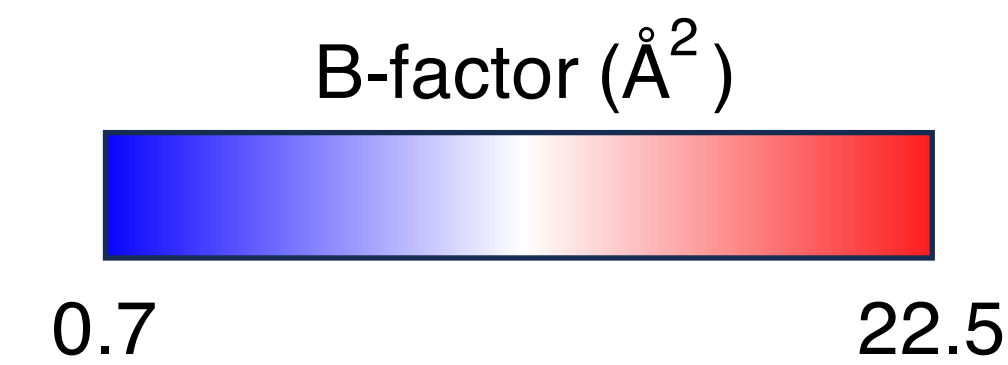

c) *Tdes*

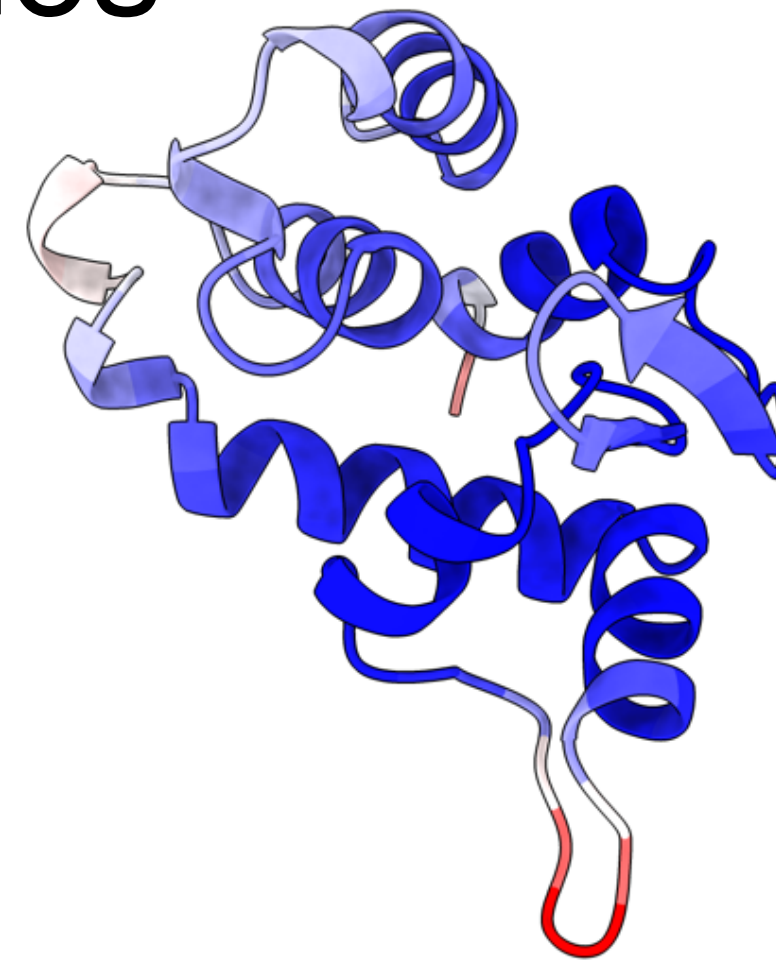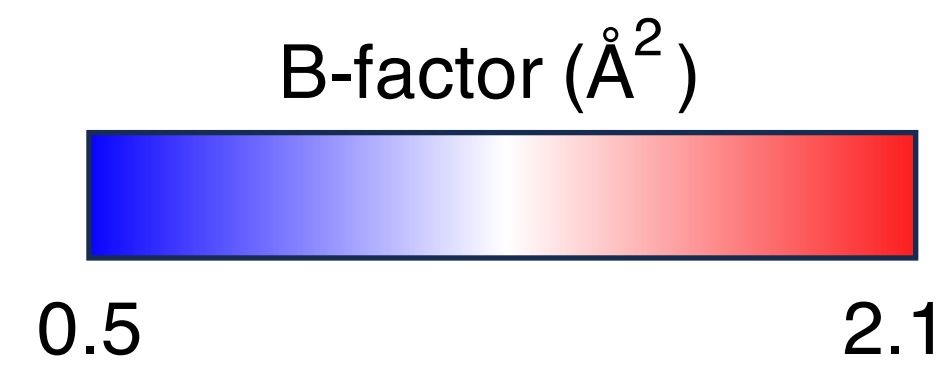

d) *Tsap*

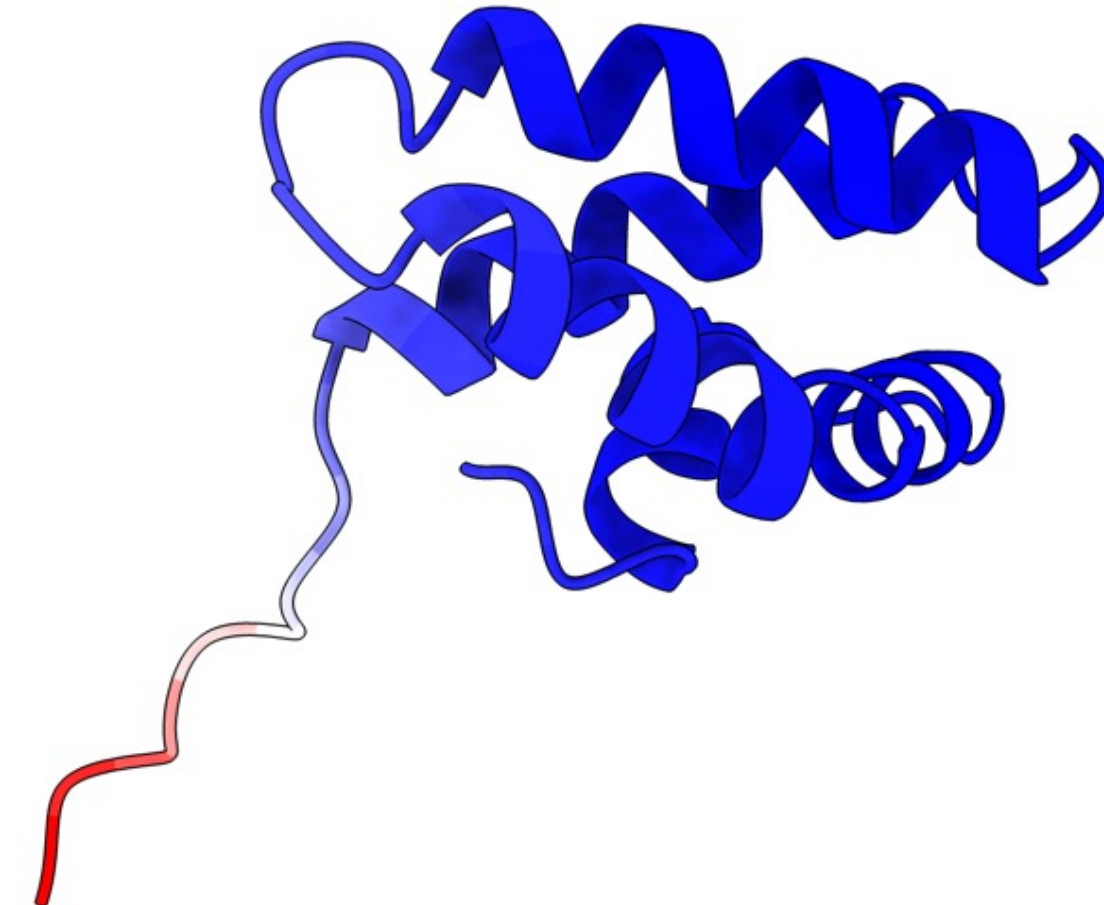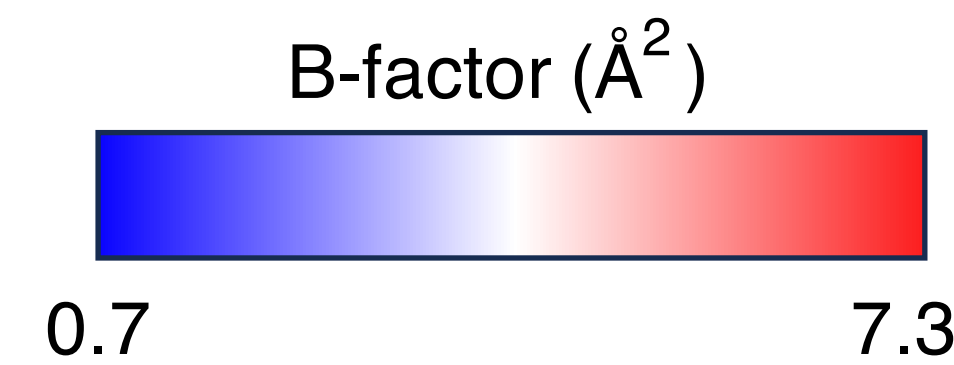

e) *Tscp*

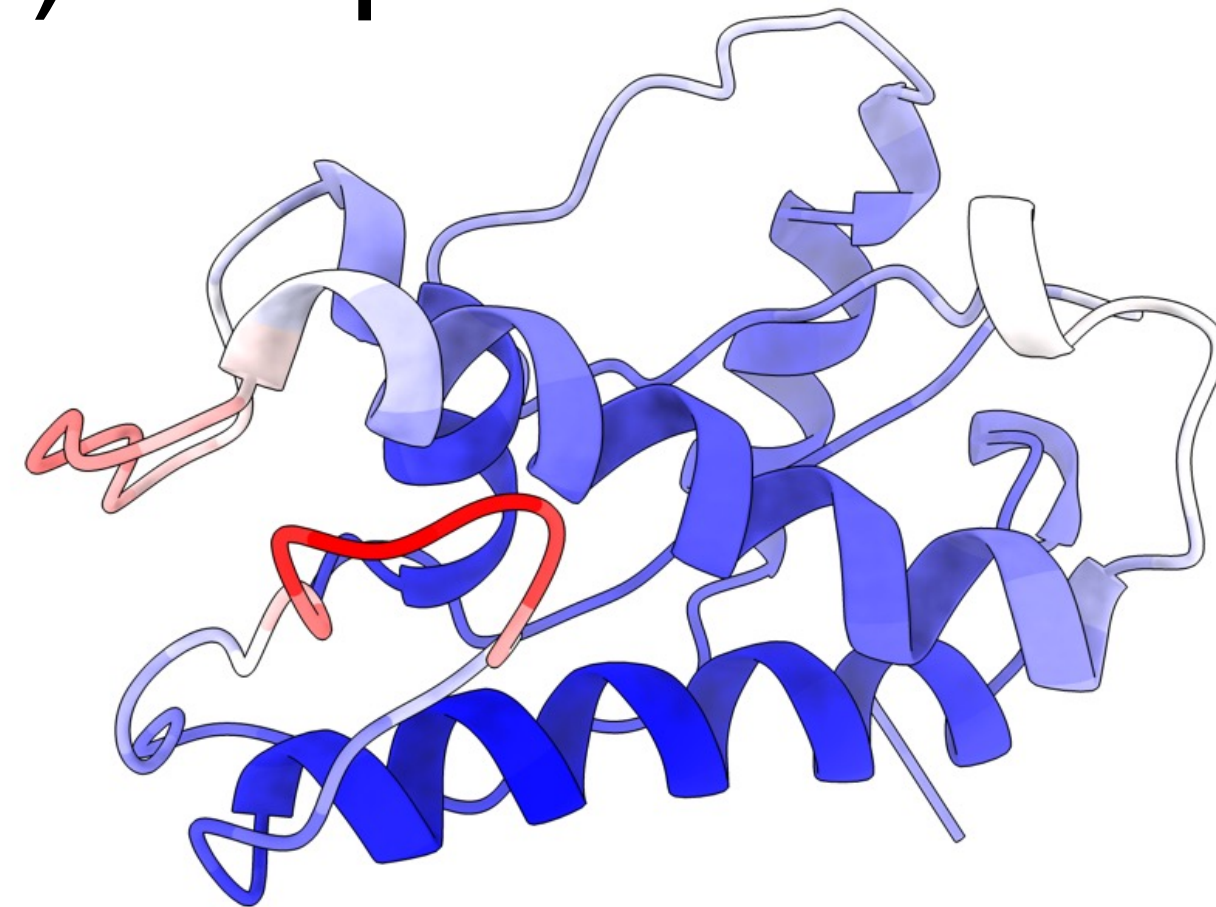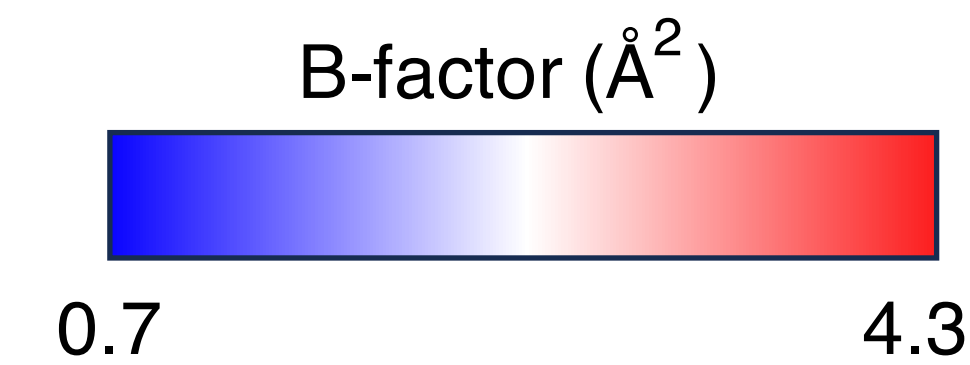

Supplement: Supplementary file 8 — Supplementary material [file mmc4.pdf]

a)  $H_{met}$

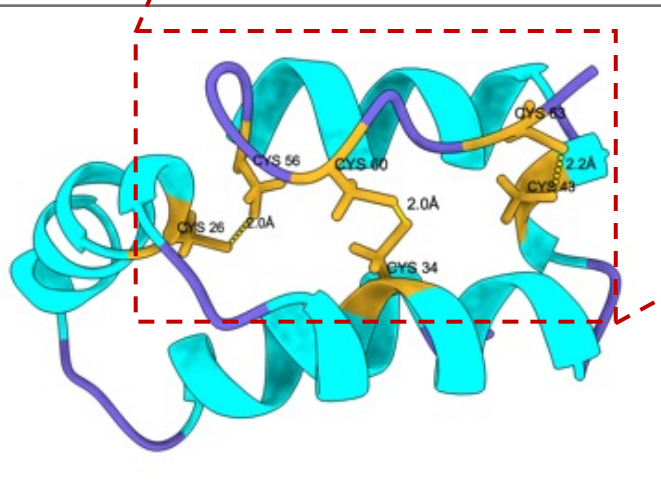

b)  $T_{des}$

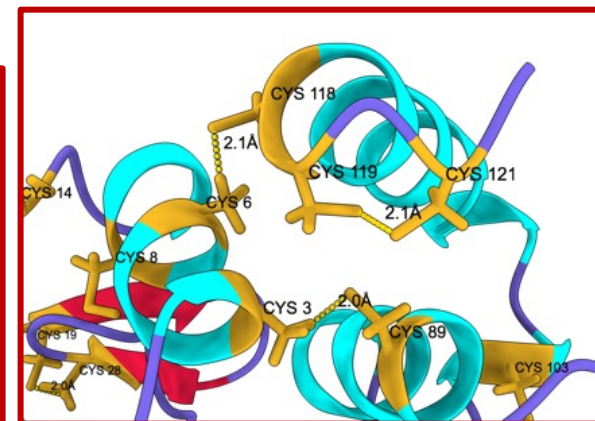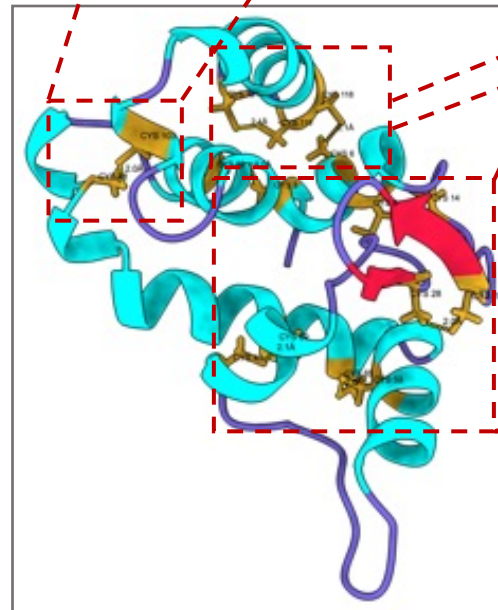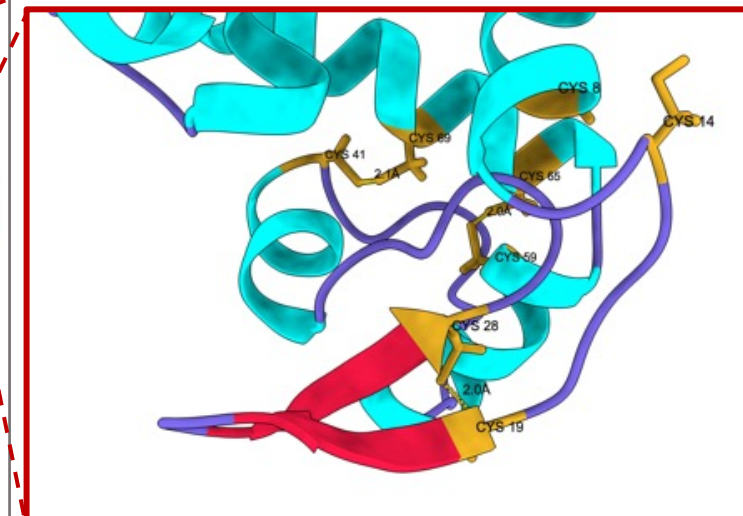

c)  $T_{sap}$

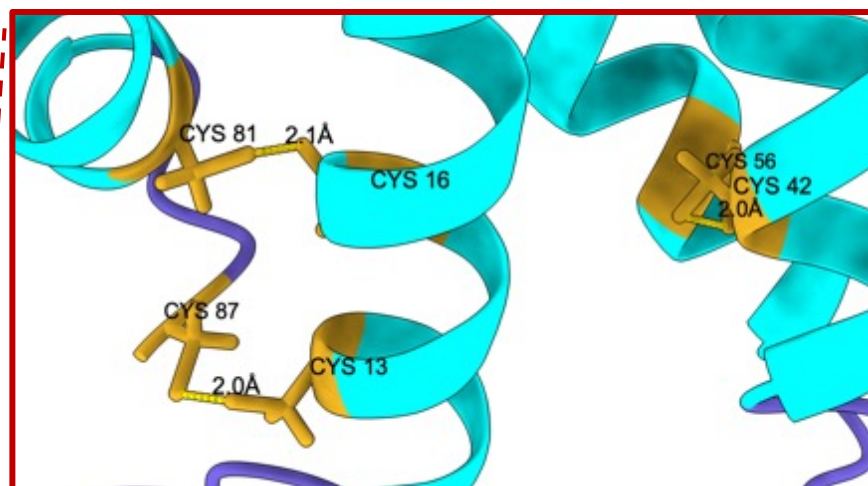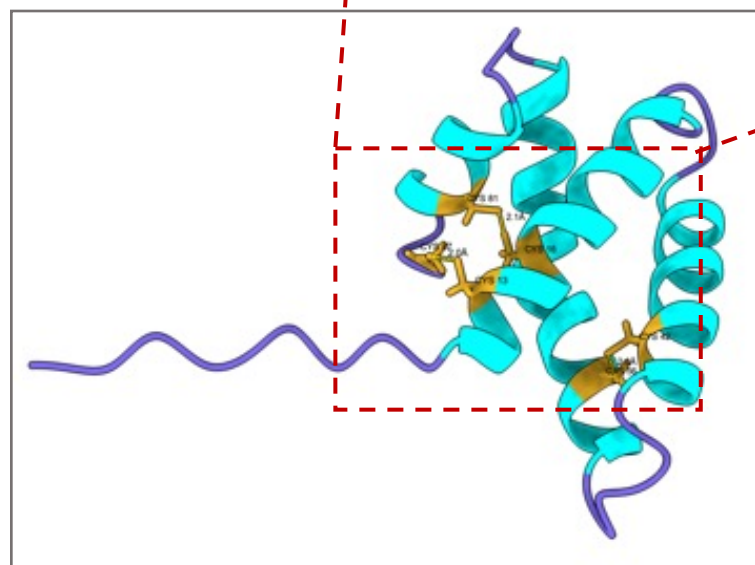

d)  $T_{scp}$

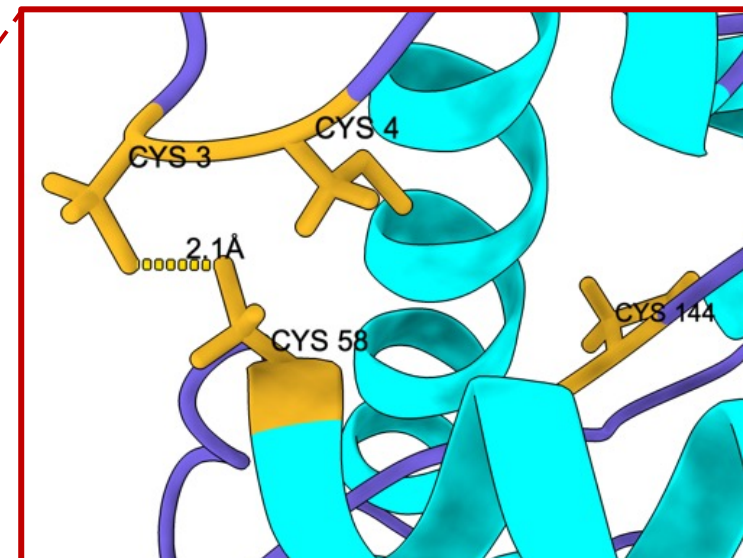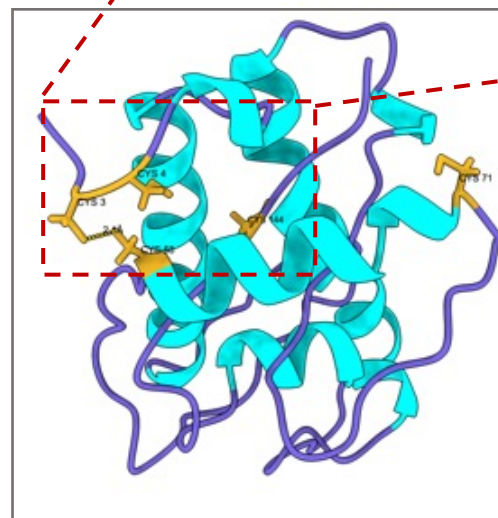

Supplement: Supplementary file 9 — Supplementary material [file mmc5.pdf]

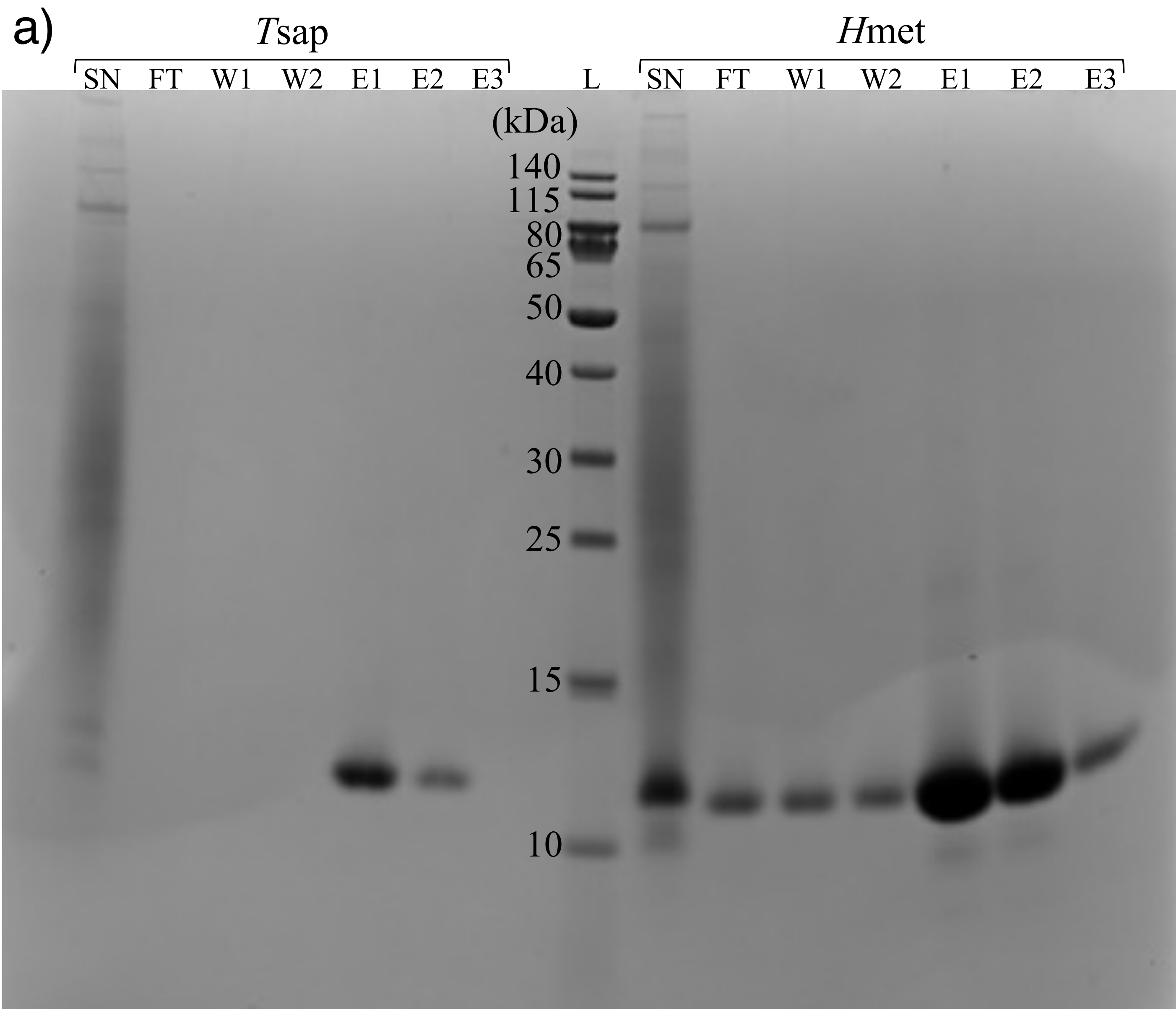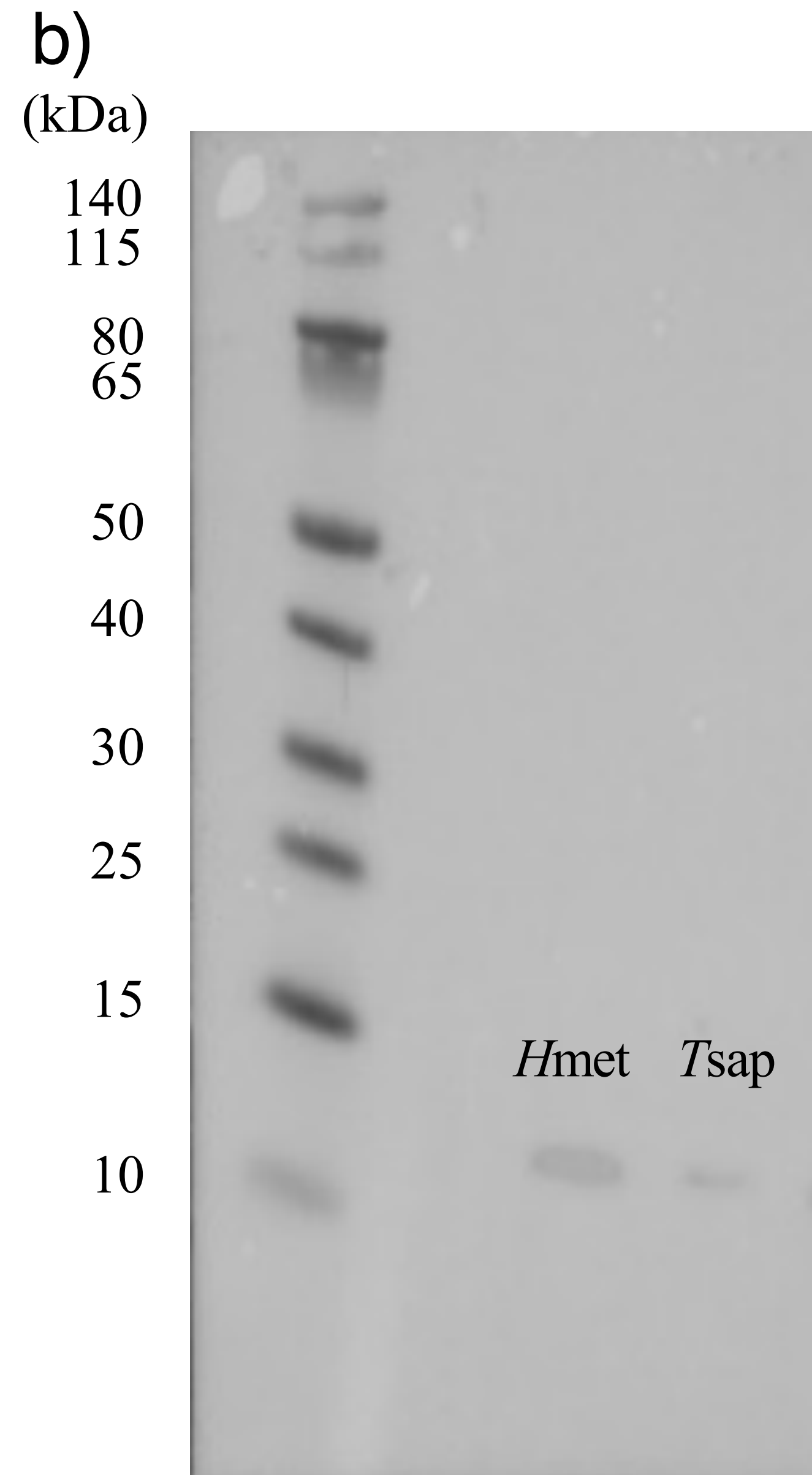

Supplement: Supplementary file 12 — Supplementary material [file mmc8.pdf]

# U-Net Segmentation

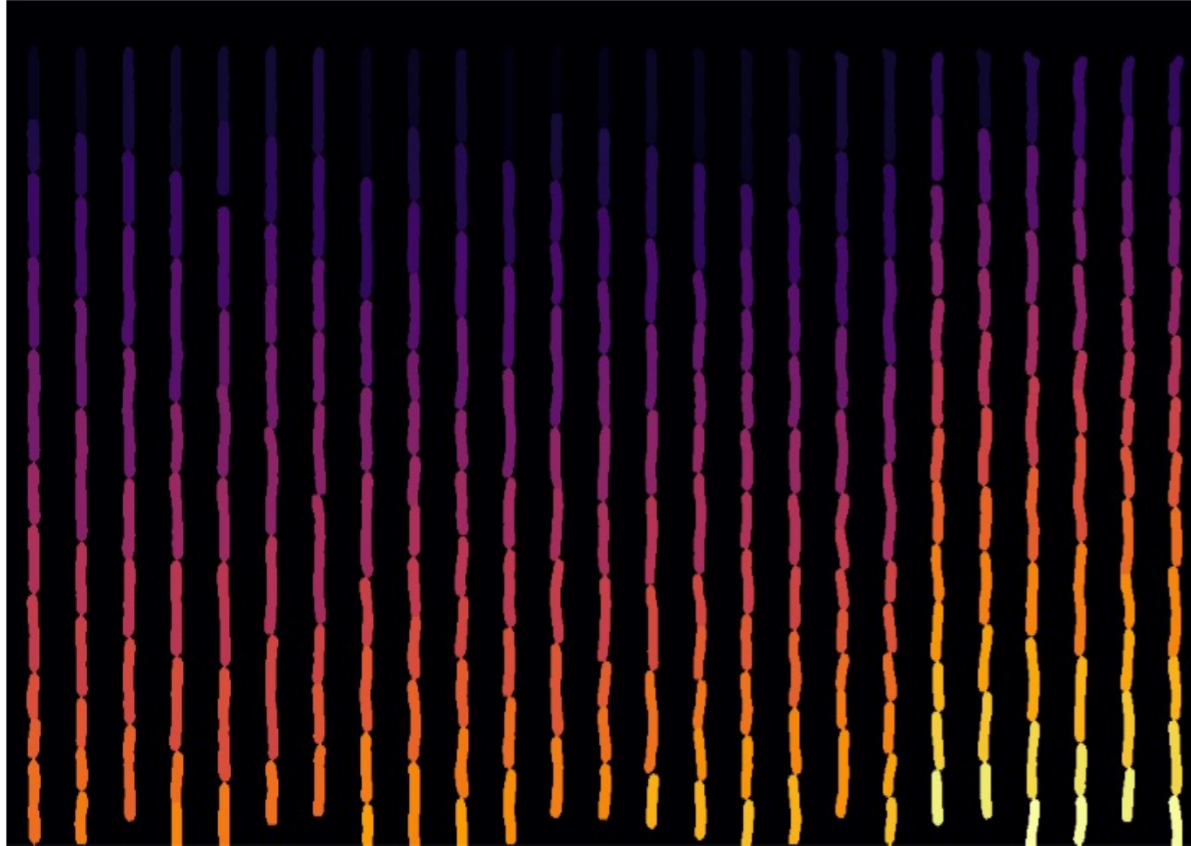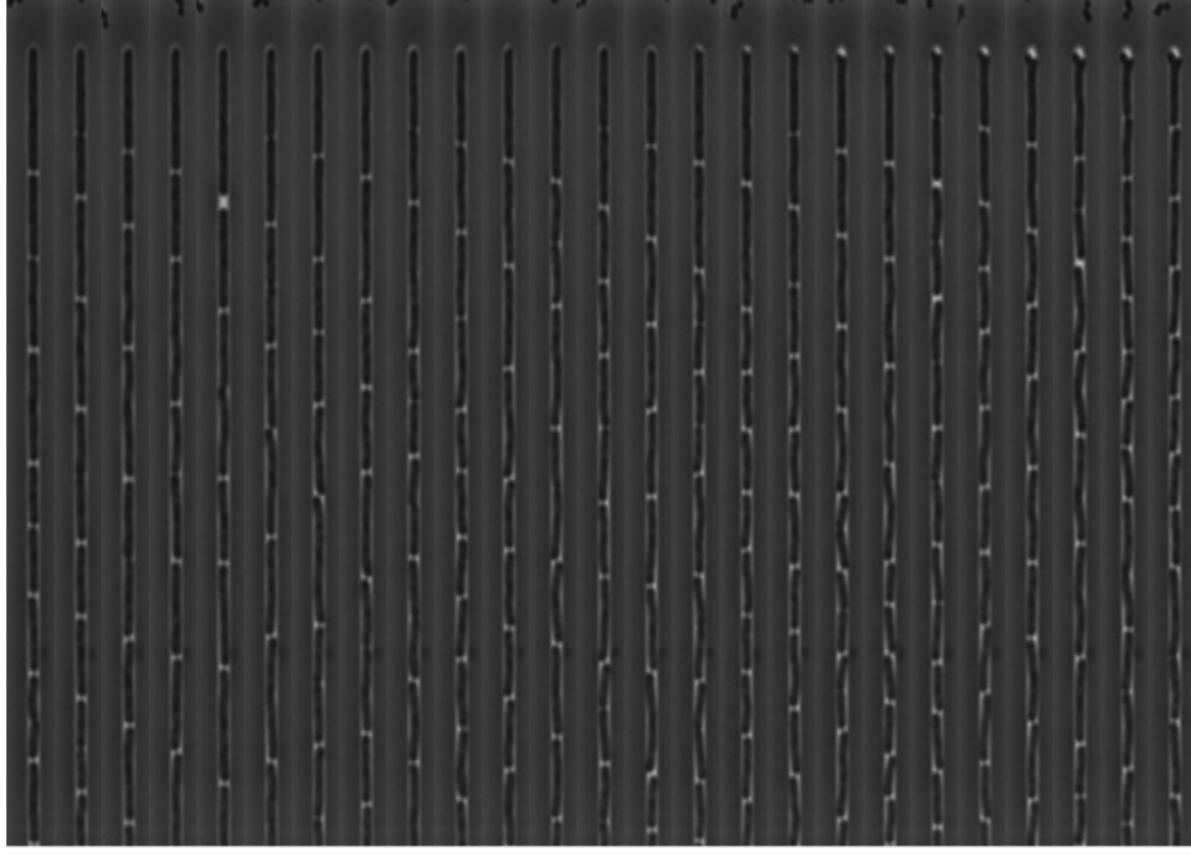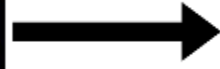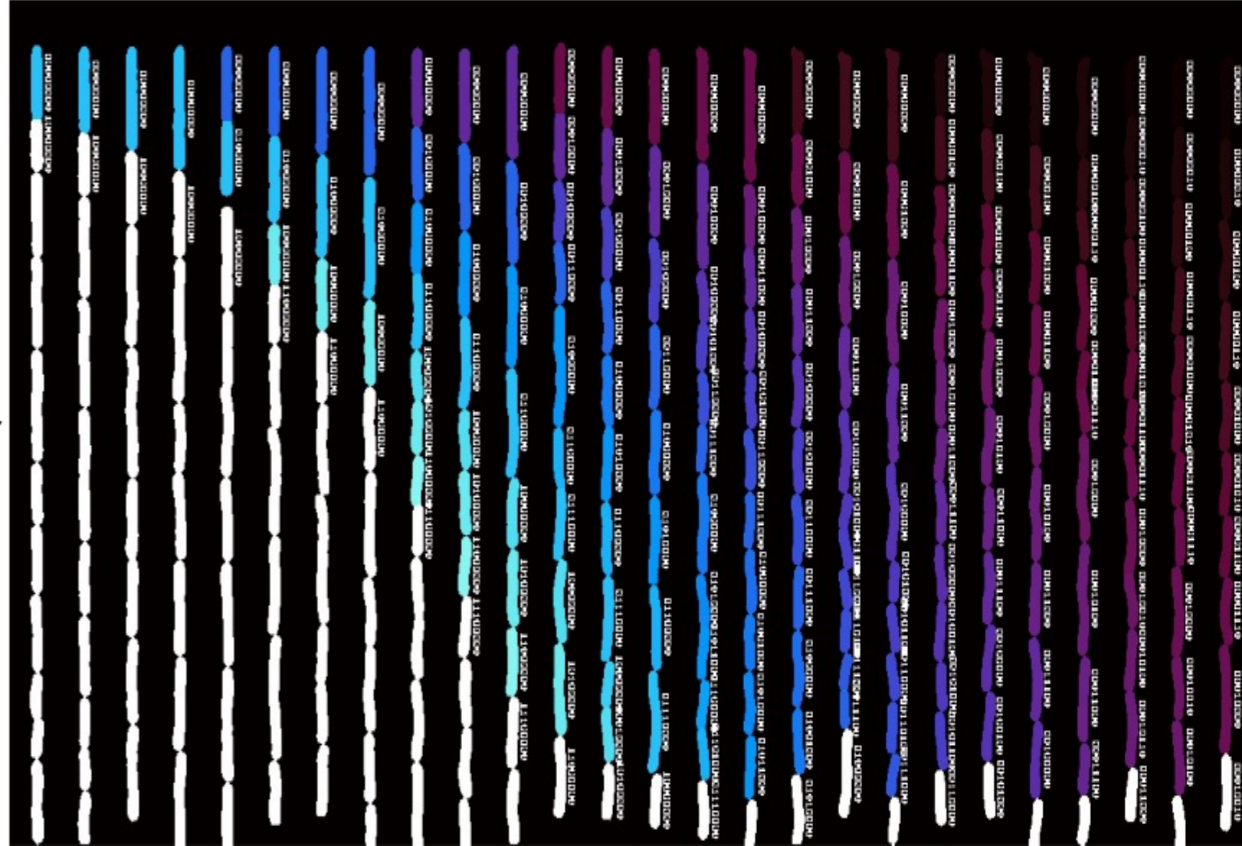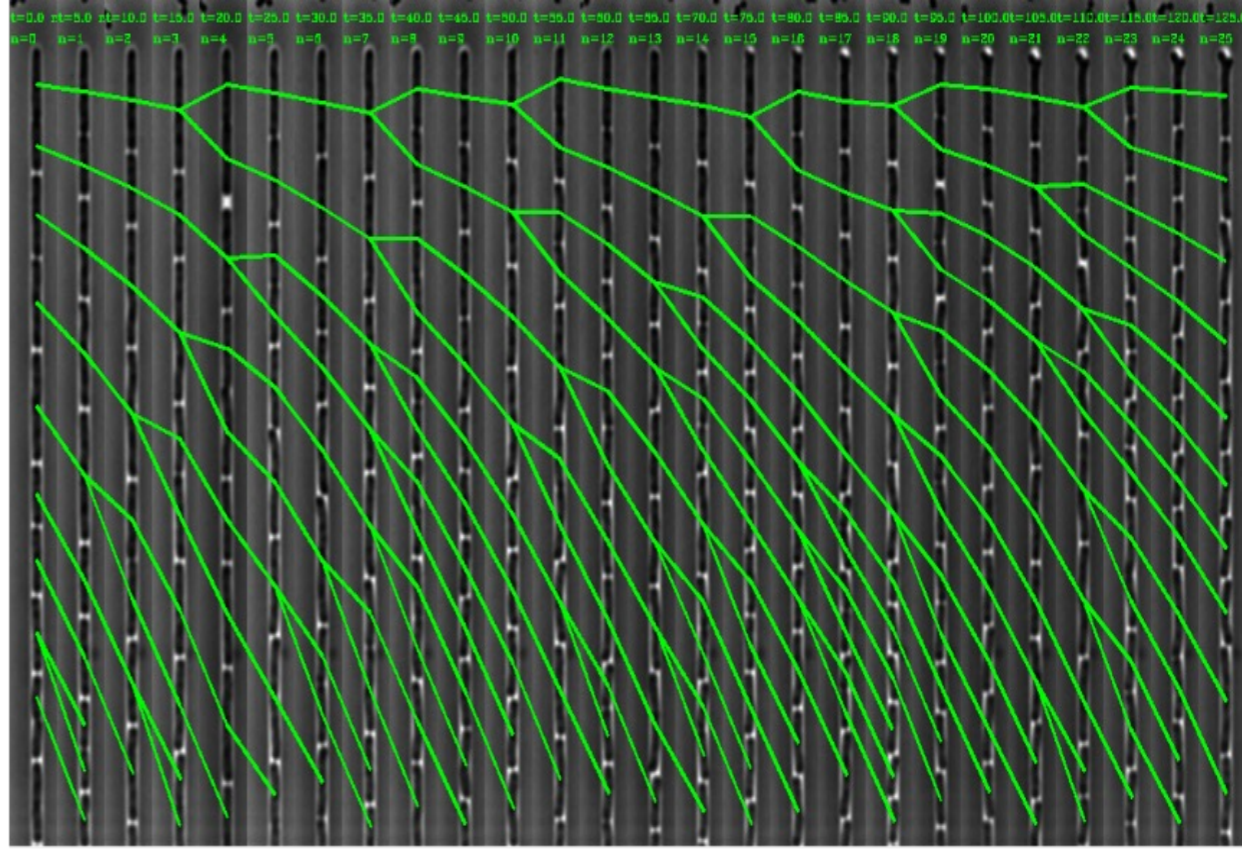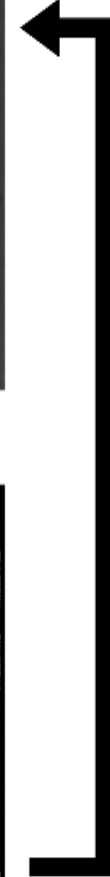

Supplement: Supplementary file 13 — Supplementary material [file mmc9.pdf]
