# Supplementary material for: Structural and functional analyses of nematode-derived antimicrobial peptides support the occurrence of direct mechanisms of worm-microbiota interactions
Source: Comput Struct Biotechnol J. 2024 Apr 10;23:1522–33. doi: 10.1016/j.csbj.2024.04.019 (PMC11021794; doi:10.1016/j.csbj.2024.04.019)
Supplement: Supplementary file 6 — Supplementary material [file mmc2.docx]

|  | **Species** | **Life cycle stage** | **No. of proteins/peptides identified** | **No. of proteins/peptides analysed in the current study** | **Reference** | **DOI** |
| --- | --- | --- | --- | --- | --- | --- |
| ESP |  |  |  |  |  |  |
|  | *Ascaris suum* | L4 | 58 | 57 | [77] | https://doi.org/10.1371/journal.pntd.0002467 |
|  |  | Adult | 175 | 175 | [78] | https://doi.org/10.1371/journal.pntd.0002939 |
|  |  |  |  |  |  |  |
|  | *Haemonchus contortus* | L3 | 311 | 311 | [79] | https://doi.org/10.1016/j.jprot.2019.05.003 |
|  |  | L4 | 535 | 535 | [79] | https://doi.org/10.1016/j.jprot.2019.05.003 |
|  |  | Adult | 1028 | 1028 | [79] | https://doi.org/10.1016/j.jprot.2019.05.003 |
|  |  |  |  |  |  |  |
|  | *Heligmosomoides polygyrus* | L4 | 590 | 590 | [80] | https://doi.org/10.1186/s13071-021-04613-9 |
|  |  |  |  |  |  |  |
|  | *Nippostrongylus brasiliensis* | L3 | 52 | 31 | [81] | https://doi.org/10.1074/mcp.M114.038950 |
|  |  | Adult | 261 | 162 | [81] | https://doi.org/10.1074/mcp.M114.038950 |
|  |  |  |  |  |  |  |
|  | *Strongyloides ratti* | Adult and larvae (mixed) | 586 | 380 | [82] | https://doi.org/10.1074/mcp.M111.010157 |
|  |  |  |  |  |  |  |
|  | *Teladorsagia circumcincta* | Adult | 423 | 423 | [24] | https://doi.org/10.1186/s13071-022-05443-z |
|  |  |  |  |  |  |  |
|  | *Toxocara canis* | L3 | 83 | 83 | [83,84] | <https://doi.org/10.1016/j.molbiopara.2016.09.002>  https://doi.org/10.1016/j.vetpar.2018.06.015 |
|  |  |  |  |  |  |  |
|  | *Trichuris suis* | Adult and larvae (mixed) | 354 | 354 | [85] | https://doi.org/10.1038/s41598-018-34174-4 |
|  |  |  |  |  |  |  |
| EV |  |  |  |  |  |  |
|  | *Ascaris suum* | Adult | 268 | 268 | [25] | https://doi.org/10.1080/20013078.2019.1578116 |
|  |  |  |  |  |  |  |
|  | *Heligmosomoides polygyrus* | Adult | 361 | 361 | [86] | https://doi.org/10.1038/ncomms6488 |
|  |  |  |  |  |  |  |
|  | *Nippostrongylus brasiliensis* | Adult | 81 | 81 | [87] | https://doi.org/10.3389/fimmu.2018.00850 |
|  |  |  |  |  |  |  |
|  | *Teladorsagia circumcincta* | L3 | 12 | 12 | Unpublished | DOI: 10.17632/hzrcpjh6y7.1 |
|  |  | L4 | 48 | 48 | Unpublished | DOI: 10.17632/hzrcpjh6y7.1 |
|  |  | L4 | 85 | 85 | [27] | https://doi.org/10.1016/j.vetpar.2016.03.008 |
|  |  | Adult | 67 | 67 | Unpublished | DOI: 10.17632/hzrcpjh6y7.1 |
|  |  | Adult | 55 | 55 | [24] | https://doi.org/10.1186/s13071-022-05443-z |

**References**

[77]Wang T., Van Steendam K., Dhaenens M., Vlaminck J., Deforce D., et al. Proteomic analysis of the excretory-secretory products from larval stages of *Ascaris suum* reveals high abundance of glycosyl hydrolases. PLoS Negl Trop Dis 2013;7:e2467. doi:10.1371/journal.pntd.0002467.

[78]Chehayeb J.F., Robertson A.P., Martin R.J., Geary T.G. Proteomic analysis of adult *Ascaris suum* fluid compartments and secretory products. PLoS Negl Trop Dis 2014;8:e2939. doi:10.1371/journal.pntd.0002939.

[79]Wang T., Ma G., Ang C.S., Korhonen P.K., Koehler A.V., et al. High throughput LC-MS/MS-based proteomic analysis of excretory-secretory products from short-term in vitro culture of *Haemonchus contortus*. J Proteom 2019;204:103375. doi:10.1016/j.jprot.2019.05.003.

[80]Maruszewska-Cheruiyot M., Szewczak L., Krawczak-Wójcik K., Głaczyńska M., Donskow-Łysoniewska K. The production of excretory-secretory molecules from *Heligmosomoides polygyrus bakeri* fourth stage larvae varies between mixed and single sex cultures. Parasit Vector 2021;14:106. <https://doi.org/10.1186/s13071-021-04613-9>.

[81]Sotillo J., Sanchez-Flores A., Cantacessi C., Harcus Y., Pickering D., et al. Secreted proteomes of different developmental stages of the gastrointestinal nematode *Nippostrongylus brasiliensis*. Mol Cell Proteom 2014;13:2736–2751. doi:10.1074/mcp.M114.038950.

[82]Soblik H., Younis A.E., Mitreva M., Renard B.Y., Kirchner M., et al. Life cycle stage-resolved proteomic analysis of the excretome/secretome from *Strongyloides ratti*—identification of stage-specific proteases. Mol Cell Proteom 2011;10:M111.010157. doi:10.1074/mcp.M111.010157.

[83]Sperotto R.L., Kremer F.S., Aires Berne M.E., Costa de Avila L.F., da Silva Pinto L., et al. Proteomic analysis of *Toxocara canis* excretory and secretory (TES) proteins. Mol Biochem Parasitol 2017;211:39–47. doi:10.1016/j.molbiopara.2016.09.002.

[84]da Silva M.B., Urrego A.J.R., Oviedo Y., Cooper P.J., Pacheco L.G.C., et al. The somatic proteins of *Toxocara canis* larvae and excretory-secretory products revealed by proteomics. Vet Parasitol 2018;259:25–34. doi:10.1016/j.vetpar.2018.06.015.

[85]Leroux L.P., Nasr M., Valanparambil R., Tam M., Rosa B.A., et al. Analysis of the *Trichuris suis* excretory/secretory proteins as a function of life cycle stage and their immunomodulatory properties. Sci Rep 2018;8:15921. doi:10.1038/s41598-018-34174-4.

[86]Buck A.H., Coakley G., Simbari F., McSorley H.J., Quintana J.F., et al. Exosomes secreted by nematode parasites transfer small RNAs to mammalian cells and modulate innate immunity. Nat Commun 2014;(1):5488. doi:10.1038/ncomms6488.

[87]Eichenberger R.M., Ryan S., Jones L., Buitrago G., Polster R., et al. Hookworm secreted extracellular vesicles interact with host cells and prevent inducible colitis in mice. Front Immunol 2018;9:850. doi:10.3389/fimmu.2018.00850.
