# Supplementary material for: Structural and functional analyses of nematode-derived antimicrobial peptides support the occurrence of direct mechanisms of worm-microbiota interactions
Source: Comput Struct Biotechnol J. 2024 Apr 10;23:1522–33. doi: 10.1016/j.csbj.2024.04.019 (PMC11021794; doi:10.1016/j.csbj.2024.04.019)
Supplement: Supplementary file 11 — Supplementary material [file mmc7.pdf]

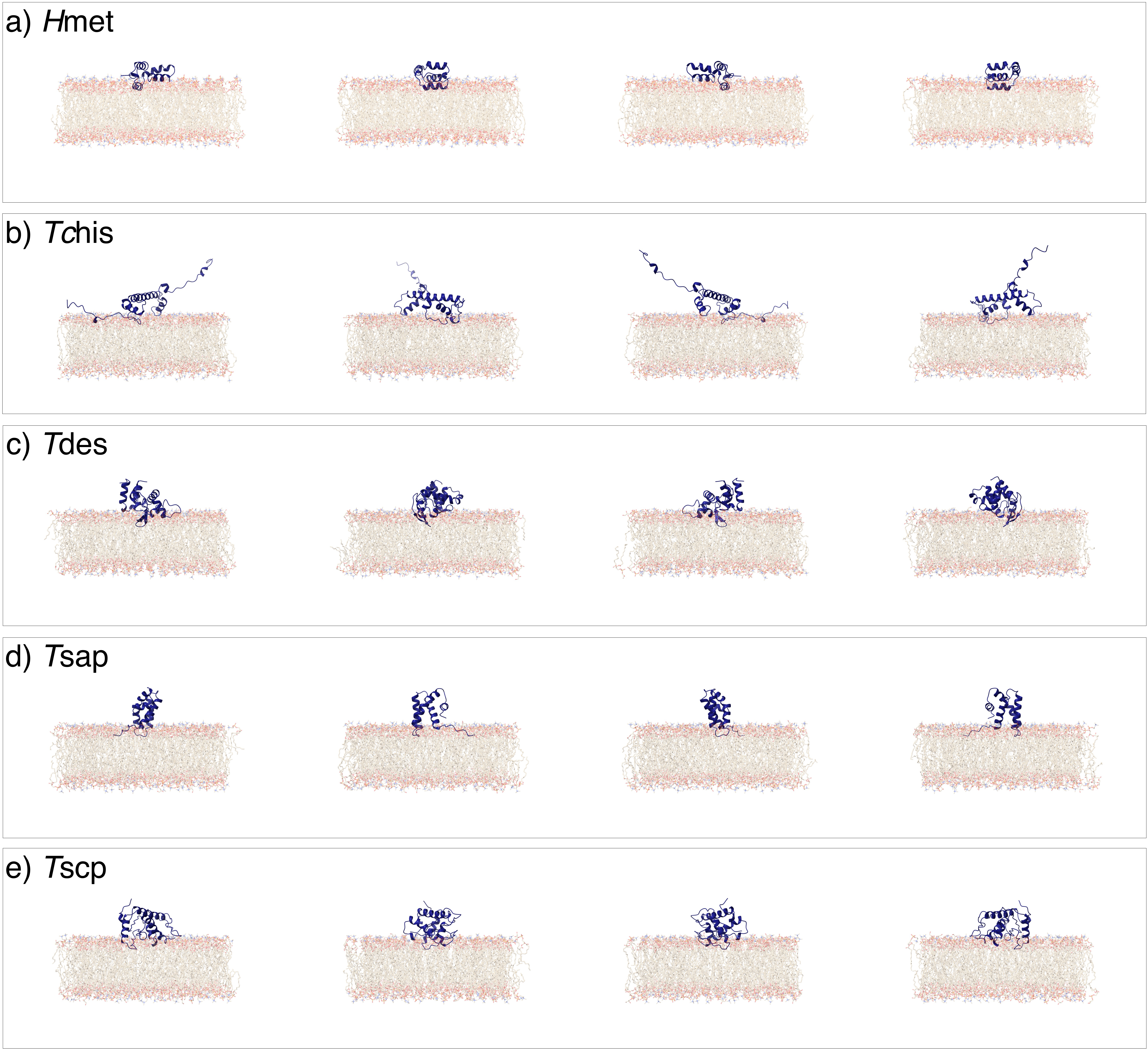

a) *H*met
b) *Tc*his
c) *T*des
d) *T*sap
e) *T*scp
